# MicroRNA Post-transcriptional Regulation of the NLRP3 Inflammasome in Immunopathologies

**DOI:** 10.3389/fphar.2019.00451

**Published:** 2019-05-01

**Authors:** Gulcin Tezcan, Ekaterina V. Martynova, Zarema E. Gilazieva, Alan McIntyre, Albert A. Rizvanov, Svetlana F. Khaiboullina

**Affiliations:** ^1^Institute of Fundamental Medicine and Biology, Kazan Federal University, Kazan, Russia; ^2^Centre for Cancer Sciences, Faculty of Medicine and Health Sciences, University of Nottingham, Nottingham, United Kingdom; ^3^Department of Microbiology and Immunology, University of Nevada, Reno, Reno, NV, United States

**Keywords:** NLRP3, inflammasome, microRNA, inflammation, disease

## Abstract

Inflammation has a crucial role in protection against various pathogens. The inflammasome is an intracellular multiprotein signaling complex that is linked to pathogen sensing and initiation of the inflammatory response in physiological and pathological conditions. The most characterized inflammasome is the NLRP3 inflammasome, which is a known sensor of cell stress and is tightly regulated in resting cells. However, altered regulation of the NLRP3 inflammasome is found in several pathological conditions, including autoimmune disease and cancer. *NLRP3* expression was shown to be post-transcriptionally regulated and multiple miRNA have been implicated in post-transcriptional regulation of the inflammasome. Therefore, in recent years, miRNA based post-transcriptional control of NLRP3 has become a focus of much research, especially as a potential therapeutic approach. In this review, we provide a summary of the recent investigations on the role of miRNA in the post-transcriptional control of the NLRP3 inflammasome, a key regulator of pro-inflammatory IL-1β and IL-18 cytokine production. Current approaches to targeting the inflammasome product were shown to be an effective treatment for diseases linked to NLRP3 overexpression. Although utilizing NLRP3 targeting miRNAs was shown to be a successful therapeutic approach in several animal models, their therapeutic application in patients remains to be determined.

## Inflammasome

### Structure

In 2002, the ground breaking work published by Martinon et al. (2002) has demonstrated the role of the inflammasome, a multi-protein complex, in the activation of pro-inflammatory caspases. The authors described the multistep process of the inflammasome assembly which is initiated by the detection of pathogen-associated molecular patterns (PAMPs) or danger signals released by damaged cells (Duncan et al., 2009; Ichinohe et al., 2010; Costa et al., 2012). Several inflammasome sensors were later identified including the nucleotide-binding oligomerization domain (NOD) like receptors (NLRs), the absent in melanoma-2 like receptors (ALRs) and pyrin (Ting et al., 2008). In the past decade our understanding of NLR containing inflammasomes structure and assembly mechanisms has advanced considerably, largely due to their potential involvement in pathogenesis of several diseases (Hoffman et al., 2001; Alexander So and Borbála Pazár, 2010; Song et al., 2017). NLRs contain three domains, an N-terminal domain, a NOD, and a C-terminal leucine-rich repeat (LRR) (Inohara and Nunez, 2003). The N-terminal domain contains a caspase recruitment domain (CARD) or pyrin domain (PYD), which function to interact with downstream molecules, such as apoptosis-associated speck-like protein containing (ASC) (Inohara and Nunez, 2003; Schroder and Tschopp, 2010). The NOD domain is linked to LRR detecting PAMPs (Boekhout et al., 2011). Upon sensing PAMPs, the NLRs polymerize followed by the interaction between the PYD or CARD domains of LLR and ASC (Stutz et al., 2013). Once activated the inflammasome adopts a wheel-like structure (Hu et al., 2015), where CARD–CARD interactions are essential for recruiting pro-caspase 1 (PC1) into close proximity with the complex (Faustin et al., 2007). PC1 becomes proteolytically cleaved by the CARD domain releasing an active caspase 1 (AC1) p10/p20 tetramer (Martinon et al., 2002; Kanneganti et al., 2006; Boucher et al., 2018).

### NLR Inflammasomes

This family of inflammasomes includes two subgroups based on the presence of CARD or pyrin in the N terminus. Only nucleotide-binding domain leucine-rich repeats proteins (NLRP)1, NLRP3, and NLRC4 were shown to form inflammasomes that produce AC1 (Mao et al., 2014). In contrast, NLRP6, NLRP9b, and NLRP12 are believed to form inflammasomes, but their roles as inflammasome sensors are less recognized (Anand et al., 2012; Vladimer et al., 2012; Zhu et al., 2017).

#### NLRP1

NLRP1 was the first identified cytosolic receptor capable of forming active inflammasomes (Martinon et al., 2002). PYD, NBD, and LRR domains, a ‘function-to find’ domain (FIIND) and a C-terminal CARD are the structural components of NLRP1 (Jin et al., 2013b). Our knowledge of NLRP1 function comes largely from studying animal models. It appears that NLRP1 senses and protects against microbial pathogens, as was shown using a mouse model of *Bacillus anthracis* and *Shigella flexneri* infection (Boyden and Dietrich, 2006; Sandstrom et al., 2019). Additionally, NLRP1 inflammasomes facilitate parasite clearance and protection as demonstrated in *Toxoplasma gondii* infection in mouse and rat models (Cirelli et al., 2014; Gorfu et al., 2014). The clinical relevance of NLRP1 inflammasomes against *Toxoplasma gondii* is also evident in individuals with specific single-nucleotide polymorphisms in the *NLRP1* gene, which are linked to congenital toxoplasmosis (Witola et al., 2011).

Aberrant activation of NLRP1 is linked to a pathogenesis of inflammatory diseases. Polymorphisms in the *NLRP1* gene are linked to Crohn’s disease, rheumatoid arthritis (RA) and systemic sclerosis (Finger et al., 2012). Although the mechanism of NLRP1 activation remains largely unknown, recently, the failure of inflammasome inhibition by dipeptidyl dipeptidase 9 (DDP9), linked to antigen processing (Zhong et al., 2018), was demonstrated to play role in pathogenesis of an autoimmune diseases (Zhong et al., 2018). The authors identified that a single mutation in the FIIND domain of NLRP1 abrogates binding to DPP9, triggering over activation of the inflammasome in autoinflammatory disease AIADK.

#### NLRC4

Similar to NLRP1, NLRC4 establishes protection against infectious pathogens (Mariathasan et al., 2004; Franchi et al., 2006; Zhao et al., 2011). In the absence of stimulus, NLRC4 remains inactive, where its NBD domain retains a closed conformation by binding to the winged helix domain (Tenthorey et al., 2014). NLRC4 activation is indirect, and it requires NLR family apoptosis inhibitory proteins (NAIPs) for the initial sensing of the microbial ligand (Rayamajhi et al., 2013; Yang et al., 2013; Kortmann et al., 2015). NAIPs trigger NLRC4 oligomerization, which is essential for inflammasome activation (Hu et al., 2015). Loss of the control over NLRC4 expression and subsequent production of AC1 and release of IL-1β by macrophages was suggested to play role in the pathogenesis of inflammasome linked autoinflammation (von Moltke et al., 2012; Canna et al., 2014). Also, a missense mutation in the NLRC4 gene was found in familial cold autoinflammatory syndrome (Kitamura et al., 2014). Multiple mutations in NLRC4 were identified in several autoinflammatory diseases including atopic dermatitis, periodic fever, and fatal or near-fatal episodes of autoinflammation (Nakamura et al., 2010; Canna et al., 2014; Bonora et al., 2015). These data suggest that NLRC4 plays role in protection against microbial pathogens and autoinflammation.

#### NLRP6

NLRP6 is an inflammasome which plays a role in gut health and maintaining mucosal response to pathogens (Elinav et al., 2011; Anand et al., 2012). A microbial metabolite, taurine, was identified as an NLRP6 activator (Levy et al., 2015). The NLRP6-taurite axis appears to be essential for the health of the gut mucosa and microbiome. Taurite produced by the normal microbiota activates NLRP6 which prevents dysbacteriosis by promoting production of antimicrobial peptides (Levy et al., 2015).

#### NLRP12

NLRP12 is intracellular protein expressed in cells of myeloid lineages (Arthur et al., 2010). NLRP12 inflammasome expression can be downregulated by microbial ligands (Williams et al., 2005; Lich et al., 2007) via canonical and non-canonical inhibition of NF-κB (Zaki et al., 2011; Allen et al., 2012). Several ligands were identified as NLRP12 activators including microbes (Allen et al., 2012; Vladimer et al., 2012).

### ALR Family Inflammasomes

ALR family inflammasomes contain an N-terminal PYD and a C-terminal hematopoietic interferon-inducible nuclear protein with 200-amino acid repeat (HIN200) domain (Cridland et al., 2012). ALR inflammasomes sense cytosolic double stranded DNA (dsDNA) (Burckstummer et al., 2009; Ferreri et al., 2010). Absent in melanoma 2 (AIM2) is the best characterized member of ALR inflammasomes. Similar to other ALR family members, AIM2 senses dsDNA; however, it appears that dsDNA recognition is independent of nucleic acid sequence as it could bind to both, microbial and host genomic material (Jin et al., 2012). dsDNA binding to HIN200 causes its dissociation from the PYD domain (Jin et al., 2012), allowing the freed PYD domain to interact with ASC, and inflammasome assembly (Jin et al., 2013c). AIM2 was implicated in the recognition of microbial, host and tumor derived dsDNA (Davis B.K. et al., 2011; Choubey, 2012; Dihlmann et al., 2014).

### Pyrin

Pyrin is an inflammasome sensor complex, which contains a N-terminal PYD, central B-box and coiled-coil domain, and a C-terminal B30.2/SPRY domain (Heilig and Broz, 2018). Pyrin was proposed to sense the changes in actin cytoskeletal dynamics as it was found co-localized with stress actin filaments (Xu et al., 2014). Microtubules promote ASC recruitment and the oligomerization (Gao et al., 2016); however, the physiological relevance of this interaction remains largely unknown. Also, microbial toxins which cause impairment of Rho GTPase activity were identified as strong activators of the pyrin inflammasome (Dumas et al., 2014; Xu et al., 2014).

Several monogenic autoinflammatory syndromes were linked to pyrin inflammasome dysregulation including familial Mediterranean fever (FMF), pyrin-associated autoinflammation with neutrophilic dermatosis, pyogenic arthritis, pyoderma gangrenosum, acne, etc. (Jamilloux et al., 2018). FMF is the most investigated pyrin inflammasome disease, characterized by repeating, self-limited, episodes of fever and polyserositis (Bernot et al., 1998). FMF is linked to a mutation in the Mediterranean Fever (MEFV) gene in a region encoding the B30.2 domain of pyrin (Omenetti et al., 2014). Also, the high prevalence of FMF within certain populations could indicate a selective pressure to preserve this mutation (Schaner et al., 2001).

### Pyroptosis

Pyroptosis is an inflammatory form of programmed cell death linked exclusively to PC1 activation (Hilbi et al., 1998). AC1 is a product of several inflammasomes: NLRP1, NLRP3, NLRC4, and AIM2. Therefore, pyroptosis is often associated with inflammasome activation. Pyroptosis differs from apoptosis in many respects including lack of DNA fragmentation (Watson et al., 2000) and sustained structural integrity of the nucleus (Zychlinsky et al., 1992). Also, pyroptosis is characterized by cell membrane pore formation, which causes cell swelling in contrast to apoptosis, where cells shrink (Fink and Cookson, 2006). Additionally, an increased intracellular osmotic pressure generates large spherical protrusions of the membrane in pyroptotic cells, which coalescence and rupture (Ona et al., 1999). Multiple studies revealed the role of pyroptosis in clearance of microbial pathogens (Sansonetti et al., 2000; Tsuji et al., 2004; Lara-Tejero et al., 2006). However, over activation of AC1 could lead to pyroptosis associated tissue damage and autoimmunity (Ona et al., 1999; Siegmund et al., 2001;Frantz et al., 2003).

## NLRP3 Inflammasomes

### Molecular Mechanism of Activation

NLRP3 is the most characterized inflammasome, and its expression is tightly regulated in resting cells (Bauernfeind et al., 2009). While NLRP3 levels in unstimulated cells are insufficient to trigger assembly of an active inflammasome complex, sensing of pathogen ligands or danger signals, triggers complex formation and pro-inflammatory cytokine production. There are multiple stimuli shown to activate NLRP3 including ATP, toxins, K^+^ efflux, reactive oxygen species and mitochondrial dysfunction (Dostert et al., 2008; Piccini et al., 2008). Upon sensing the stimulus, the nucleotide binding domain (NBD) polymerizes initiating PYD–PYD oligomerization with ASC (Lu A. et al., 2014). The CARD of ASC recruits PC1, which becomes cleaved liberating AC1 (Boucher et al., 2018). It appears that within the large family of inflammasomes, NLRP3 is the main PC1 activator (Agostini et al., 2004; Davis E.E. et al., 2011). Inflammatory AC1 liberates functional IL-1β and IL-18 (Afonina et al., 2015), pleotropic cytokines regulating inflammation and innate immune response (Garlanda et al., 2013).

**FIGURE 1:**
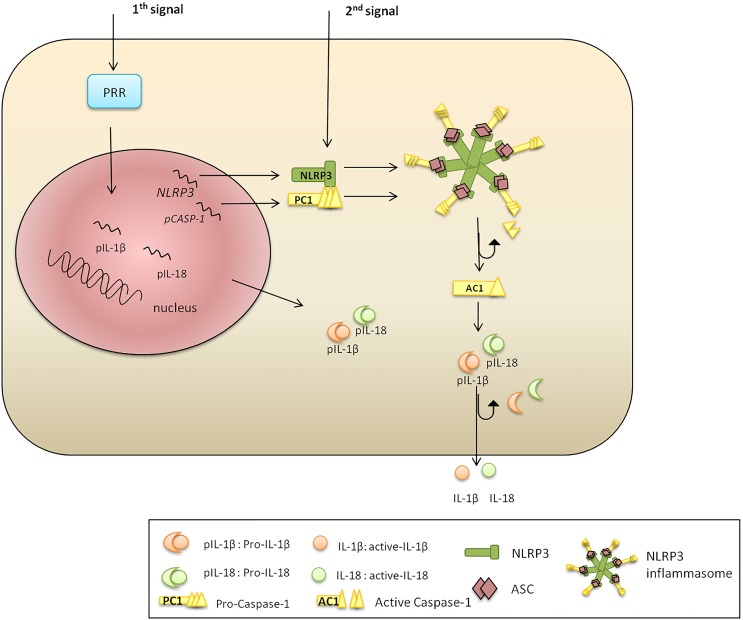
NLRP3 inflammasome activation. There are two signals required for NLRP3 inflammasome activation. Signal 1 is a priming trigger (microbial ligands, cytokines, etc.) required for the upregulation of NLRP3 and pro-IL-1β transcription and protein synthesis. Signal 2 is an activation trigger (ATP, toxins, viral RNA, etc.) which is essential for formation of an active NLRP3 inflammasome. The second stimulus promotes NLRP3, PC1, pro-IL-1β, and pro-IL-18 protein synthesis. The N-terminal NBD of NLRP3 polymerizes initiating PYD–PYD oligomerization with ASC. The CARD of ASC recruits PC1, which become cleaved liberating AC1. Inflammatory AC1 liberates functional IL-1β and IL-18, pleotropic cytokines regulating inflammation and innate immune response.

The classic pathway of NLRP3 activation requires two steps: priming and activation (Figure 1). Toll-like receptor (TLR), FAS-associated death domain protein and IL-1R ligands were identified as NLRP3 priming stimuli (Allam et al., 2014; Gurung et al., 2014; He Y. et al., 2016). The priming step includes transcriptional activation of NLRP3 via NF-κB signaling (Bauernfeind et al., 2009; Costa et al., 2012); however, it fails to initiate functional inflammasome formation, which requires a second stimulus (Jo et al., 2016). The second signal can be provided by multiple pathogen and danger associated ligands (Franchi et al., 2012; Koizumi et al., 2012), promoting the assembly of an adaptor (ASC) and PC1. The formed complex cleaves the PC1, which subsequently processes and releases functional IL-1β and IL-18 (Alnemri et al., 1996).

## Epigenetic Factors and Post-Transcriptional Mechanisms Regulating NLRP3 Inflammasome Activation

The term “epigenetic” was originally presented by Waddington (1956) to describe regulation of gene expression during the embryogenesis. Since then, definition of “epigenetic” has changed, and now refers to a stably heritable modulation of gene expression without altering DNA sequence (Berger et al., 2009). Epigenetic factors include DNA methylation at cytosine followed by guanine (CpGs) nucleotide and histone posttranslational modifications (Peschansky and Wahlestedt, 2014). Initially, epigenetic control was demonstrated in normal development and differentiation; however, its role in pathogenesis of acute and chronic inflammation has become increasingly recognized (Bayarsaihan, 2011).

### DNA Methylation

DNA methylation is dynamic and changes during the embryonic development and differentiation (Berger, 2007). It was shown that DNA methylation silences genes to ensure monoallelic expression, prevent endogenous retrovirus expression and transposon actions (Walsh et al., 1998; Bourc’his et al., 2001; Bourc’his and Bestor, 2004). DNA methylation is essential for normal cell function; however, its role in the pathogenesis of several diseases has also been confirmed (Wei et al., 2016; Vento-Tormo et al., 2017). DNA demethylation is often detected near promoters, suggesting that gene overexpression could play role in pathogenesis of many pathologies (Ryan et al., 2010; Bierne et al., 2012). NLRP3 inflammasome expression can also be regulated by changes in gene methylation status. For example, *NLRP3* gene expression is silenced in health which appears to be essential for inhibiting inflammation (Ryan et al., 2010; Bierne et al., 2012; Wei et al., 2016). However, demethylation and, subsequent, overexpression of *NLRP3* was linked to pathogenesis of cryopyrin-associated periodic syndromes (CAPS) (Vento-Tormo et al., 2017) and *Mycobacterium tuberculosis* infection (Wei et al., 2016).

### Histone Modifications

The effect of epigenetic modification of histones was studied using several inflammatory models (Bayarsaihan, 2011). Histone acetylation is essential for initiation of an activation phase of inflammation, which is characterized by the release of pro-inflammatory cytokines via CREB, mitogen-activated protein kinases (MAPKs), nuclear factor-κB (NF-κB) and signal transducer and activator of transcription (STAT) factors (Escobar et al., 2012). In contrast, histone deacetylations regulate the late, an attenuation phase of inflammation (Villagra et al., 2010). It appears that inflammasome activation can also be regulated by affecting the acetylation status of histones, as it was recently shown by Liu C.C. et al. (2018). The authors demonstrated upregulation of NLRP3 in patients diagnosed with painful neuropathy, which could be prevented by inhibition of histone acetylation.

### Non-coding RNAs

In addition to epigenetic modulation non-coding RNAs are also involved in NLRP3 regulation (Bayarsaihan, 2011), as was demonstrated in the setting of inflammation caused by microbial and viral infection (Li et al., 2010; Ryan et al., 2010; Bierne et al., 2012; Jin et al., 2013a; Chen and Ichinohe, 2015). This inflammation is post-transcriptionally regulated via non-coding RNAs targeting inflammasome components, where mRNA stability and inhibition of translation were most commonly affected (Bayarsaihan, 2011).

### Post-transcriptional Regulation of NLRP3 Inflammasomes: MicroRNA (miRNA)

**FIGURE 2:**
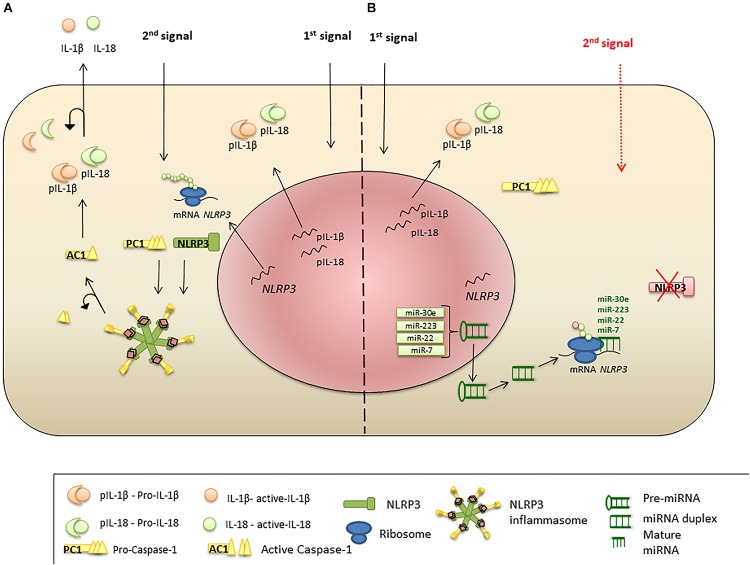
miRNA regulation of NLRP3 inflammasome expression. **(A)** Priming signal triggers NLRP3, PC1, IL-1β, and IL-18 transcription and protein synthesis. Activation signal initiates inflammasome formation and release of AC1. AC1 proteolytically cleaves pro-IL-1β and pro-IL-18, liberating active cytokines. **(B)** Suppression of NLRP3 protein translation and inflammasome formation by miRNA. Priming stimulus triggers NLRP3 transcription; however, *miR-223*, *miR-22*, *miR-30e*, and *miR-7* bind to the UTR region of NLRP3 mRNA and interrupt protein translation. Absence of NLRP3 protein leads failure of the inflammasome protein complex formation, when the second stimulus present.

MicroRNAs are endogenous conservative, single-stranded non-coding RNAs which are 19–24 nucleotides long. Usually, miRNAs are derived from transcripts with a hairpin structure and are loaded into the Argonaute protein within a silencing complex (Hutvagner and Zamore, 2002; Mourelatos et al., 2002; Bartel, 2004). The inhibitory effect of miRNAs is explained by their binding to the untranslated regions (UTRs) of transcripts which modulates the stability and translation of the target mRNA (Figure 2) (Ruvkun, 2001; Filipowicz et al., 2008; Bartel, 2009; Coll and O’Neill, 2010). miRNAs can modulate the expression of histone modifies including histone deacetylases and DNA methyltransferases resulting in modulation of histone modifications and DNA methylation (Tuddenham et al., 2006; Fabbri et al., 2007).

NLRP3 activation is tightly regulated where two signals are required to initiate functional inflammasome formation. The first signal includes cell priming with TLR ligands (Bauernfeind et al., 2009; Franchi et al., 2009). Therefore, it could be suggested that targeting TLR expression will impact the inflammasome activity. Indirect regulation of TLR expression includes modulation of the downstream pathways molecules, which has been shown in injuries, inflammation and cancer (Coll and O’Neill, 2010; Sheedy et al., 2010; Nahid et al., 2011; Anzola et al., 2018; Tan et al., 2018; Zhi et al., 2018). TLR4 ligands are the most studied priming signals of NLRP3 activation (Groslambert and Py, 2018). It was shown that the TLR ligand binding increases the level of several miRNAs, including *miR-155*, *miR-146a*, *miR-21*, and *miR-132*, which were linked to inhibition of TLR4/MyD88/NF-κB signaling (Coll and O’Neill, 2010; Sheedy et al., 2010; Nahid et al., 2011; Anzola et al., 2018; Tan et al., 2018; Zhi et al., 2018). It appears that upregulation of miRNAs is a component of a negative feedback mechanism designed to down-modulate inflammatory cytokine production after response to microbial stimuli (Ceppi et al., 2009).

A direct inhibitory effect of *let-7* family miRNAs on *TLR4* mRNA has been demonstrated (Chen et al., 2007). *Let-7* miRNA regulation of *TLR4* was shown to occur via post-transcriptional suppression (Androulidaki et al., 2009). It was suggested that *let-7* miRNA downregulation of *TLR4* could have detrimental effect on host defense against microbes, promoting microbial survival and propagation (Chen et al., 2005; Muxel et al., 2018). Post-transcriptional regulation of TLR signaling and its impact on diseases are reviewed by Nahid et al. (2011).

Active inflammasome complex formation requires a second signal, initiating substantial NLRP3 transcription (Dostert et al., 2008; Piccini et al., 2008). During this transcriptionally active phase, *NLRP3* mRNA could be regulated by miRNA, as was shown by *miR-223* (Bauernfeind et al., 2012). According to an *in silico* analysis, *miR-223* can bind to a highly conserved region of the 3′UTR of *NLRP3* mRNA and subsequently interfere with protein translation (Lewis et al., 2005). Interestingly, *miR-223* appears to be an important NLRP3 regulator in leukocytes (Bauernfeind et al., 2012; Haneklaus et al., 2012), where the miRNA levels have been shown to vary in different leukocyte subsets. For example, this miRNA was found absent in T and B lymphocytes (Bauernfeind et al., 2012; Haneklaus et al., 2012). In contrast, the *miR-223* was demonstrated in myeloid cells, where it was highest in neutrophils, followed by macrophages and dendritic cells (Bauernfeind et al., 2012). It has been suggested that this miRNA plays role in granulocyte production and regulation of inflammation (Johnnidis et al., 2008; Neudecker et al., 2017). Decreased production of pro-inflammatory cytokines such as IL-1β and IL-18 was demonstrated in cells treated with *miR-223* or its mimics (Neudecker et al., 2017; Ding Q. et al., 2018). These data suggest that *miR-223* could be a potential target for regulation of *NLRP3* expression, where increased miRNA could reduce inflammasome activation and, subsequently, abrogate the inflammation (Bauernfeind et al., 2012; Haneklaus et al., 2012).

**FIGURE 3:**
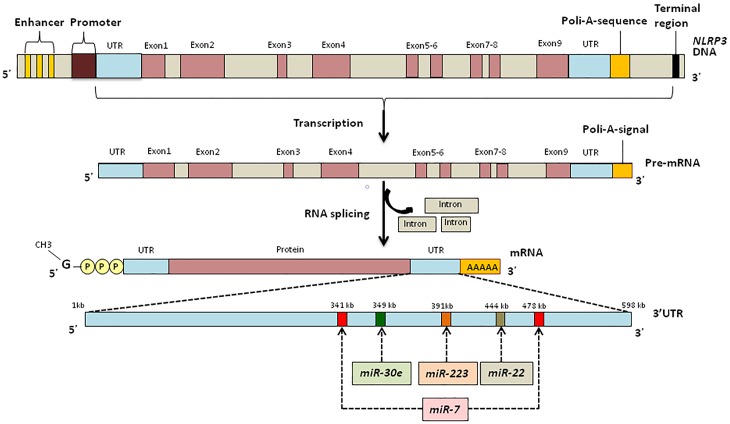
UTR binding sites of *NLRP3* for miRNAs responsible for the regulation of inflammation.

Since several miRNAs could regulate expression of a single transcript (Krek et al., 2005), it is likely that in addition to *miR-223*, other miRNAs can alter NLRP3 transcription (Figure 3).

Numerous studies have identified that pathogens, trauma and cancer can cause abnormal expression of miRNAs which impair NLPR3 inflammasome function disrupt the functional complex formation and its signaling (Table 1).

**Table 1:** Aberrant miRNA expressions linked to inflammasome related diseases.

Disease	miRNA	Regulation of miRNA	Target cell	Target gene	References
Inflammatory bowel diseases	miR-223	↑	Intestinal biopsies	NLRP3	Neudecker et al., 2017
		↑	Circulating monocytes, neutrophils		Johnnidis et al., 2008; Bauernfeind et al., 2012; Neudecker et al., 2017
		↓	Macrophages		
Rheumatoid arthritis	miR-33	↑	Macrophages	PGC1-α	Karunakaran et al., 2015; Xie Q. et al., 2018
Type 1 diabetes	miR-146a	↓	Macrophages	TLR2, TLR4	Bhatt et al., 2016; Xie Z. et al., 2018
Type 2 diabetes	miR-146a	↓	Macrophages	TLR2, TLR4	Balasubramanyam et al., 2011
Systemic lupus erythematosus	miR-23b	↓	Inflammatory lesions	TAB2, TAB3, IKK-α	Zhu et al., 2012
Parkinson’s disease	miR-7	↓	Microglia	NLRP3	Zhou Y. et al., 2016
	miR-30e	↓		NLRP3	Li D. et al., 2018
Atherosclerosis	miR-22	↓	Monocytes, macrophages	NLRP3	Huang W.Q. et al., 2017
	miR-9	↓		JAK1	Wang F. et al., 2017
	miR-30e-3p	↓		FOXO3	Li P. et al., 2018
Acute lung injury/acute respiratory distress syndrome	miR-223	↑	Ly6G+ neutrophils	NLRP3	Feng et al., 2017
Hepatocellular carcinoma	miR-223	↑	Tumor cell line	NLRP3, EPB41L3, FOXO1	Li X. et al., 2011; Kim et al., 2017
	miR-223	↓	Patient’s sera	NLRP3	Bhattacharya et al., 2016
	miR-30e	↓		NLRP3	Bhattacharya et al., 2016
Colorectal cancer	miR-223	Tumor type specific	Tumor tissue, tumor cell line	NLRP3, FoxO3a	Ju et al., 2018
	miR-22	↓		SP-1	Xia et al., 2017
Gastric cancer	miR-223	↑	Tumor tissue	NLRP3	Haneklaus et al., 2012
	miR-22	↓	Macrophages	NLRP3	Li S. et al., 2018
Oral squamous cell carcinoma	miR-223	↑	Tumor tissue	RHOB	Manikandan et al., 2016
	miR-22	↓		NLRP3	Feng et al., 2018
Cervical cancer	miR-223	↓	Tumor tissue, tumor cell line	FOXO1	Wu et al., 2012
	miR-22	↓		HDAC6	Wongjampa et al., 2018
Glioblastoma	miR-223	Controversial	Tumor tissue, tumor cell line	NFIA, PAX6	Fazi et al., 2005; Glasgow et al., 2013; Cheng et al., 2017; Ding Q. et al., 2018
	miR-22	↓		SIRT1	Li W.B. et al., 2013

#### miRNA in Regulation of Inflammasome in Infections

Inflammasome activation is an important component of infectious pathogens surveillance and antimicrobial immune and inflammatory responses. This inflammasome was shown to be activated by several bacterial pathogens including *Staphylococcus aureus*, *Salmonella typhimurium*, *Listeria monocytogenes*, *Mycobacterium*, *Streptococcus pyogenes*, *Neisseria gonorrhoeae* as well as fungi such as *Candida albicans* and *Aspergillus fumigatus* (Franchi et al., 2006; Mariathasan et al., 2006; Miao et al., 2006; Craven et al., 2009; Duncan et al., 2009; Harder et al., 2009; Hise et al., 2009; Joly et al., 2009; Munoz-Planillo et al., 2009; Broz et al., 2010; Carlsson et al., 2010; McElvania Tekippe et al., 2010; Said-Sadier et al., 2010). NAIP/NLRC4 inflammasome can protect against *Salmonella* Typhimurium and *C. rodentium* invasion by bacteria expulsion from intestinal epithelial cells together with IL-18 and eicosanoid lipid mediators release (Nordlander et al., 2014; Sellin et al., 2014; Rauch et al., 2017). It appears that NLRP3 activation is essential for establishing the inflammatory milieu in the target tissue and augmenting the phagocytic capacity of the local macrophages (Master et al., 2008; Melehani and Duncan, 2016; Cohen et al., 2018). Enhanced macrophage bactericidal activity is the most commonly identified mechanism of inflammasome antimicrobial effect (Master et al., 2008; Cohen et al., 2018). Additionally, NLRP3 activation induced death of macrophages was described as an effort to prevent microbial propagation and spread (Miao et al., 2010; Sagulenko et al., 2013). However, there is a growing body of evidence suggesting that there is a threshold of NLRP3 activity, which acts as a safeguard mechanism to prevent inflammasome over-activation. It appears that aberrant NLRP3 activation could have a detrimental effect on tissues homeostasis and compromise barrier integrity (Bortolotti et al., 2018; McKenzie et al., 2018). It is this detrimental effect of the inflammasome over-activation that is often employed by microbes to ensure spread and propagation (Duncan et al., 2009; Harder et al., 2009; Carlsson et al., 2010).

Microbial virulence factors often act as NLRP3 activators. For example, it was shown that the detrimental (to the host) role of Esx1, a membrane lysis factor of *Mycobacterium* (Stanley et al., 2003), is linked to inflammasome activation (Carlsson et al., 2010). Two virulence factors of group A *Streptococcus* (GAS), M protein and streptolysin O, were also identified as contributing into NLRP3 activation and IL-1β production (Harder et al., 2009; Valderrama et al., 2017). Both virulence factors are commonly detected in association with invasive GAS infections, including necrotizing fasciitis and toxic shock syndrome. Therefore, NLRP3 activation by virulent factors could promote microbe propagation and aid their escape from immune clearance.

Restoring the NLRP3 activation threshold could be a novel therapeutic approach for treatment of invasive infections. In this respect, miRNA may be a tool to regain control over NLRP3. It has been shown that *miR-223* expression is consistently high in NLRP3 responsive cells, suggesting the high efficacy of this miRNA in prevention of inflammasome over-activation (Bauernfeind et al., 2012). Dorhoi et al. (2013) demonstrated that *miR-223* is upregulated in the blood and lung parenchyma of patients diagnosed with tuberculosis. Also, data collected using animal models confirmed the link between deletion of *miR-223* and increased susceptibility to *Mycobacterium tuberculosum* infection (Dorhoi et al., 2013). Similarly, a protective role of *miR-223* in *Staphylococcus aureus* infection was demonstrated by Fang et al. (2016). Additionally, the effect of targeting TLR4 for NLRP3 regulation in *Listeria monocytogenes* infection was demonstrated by Schnitger et al. (2011). The authors identified that, *miR-146a* can directly inhibit TLR4 receptor expression, which can downregulate inflammasome activity (Schnitger et al., 2011).

Many viruses can activate inflammasomes, including Influenza virus, Hepatitis C virus, Herpes simplex virus-1, etc. (Delaloye et al., 2009; Ichinohe et al., 2010; Ito et al., 2012; Kaushik et al., 2012; Negash et al., 2013; Triantafilou et al., 2013a,b; Wu et al., 2013; Ermler et al., 2014; Chen and Ichinohe, 2015). Inflammasome activation appears to be essential for anti-viral protection, serving as viral genome sensors and triggering innate immune response (Muruve et al., 2008; Lupfer et al., 2015). The protective role of inflammasomes was shown in influenza virus infection as an increased viral clearance was NLRP3 dependent (Allen et al., 2009). Also, inflammasome activation improved the survival rate in an animal model of influenza (Ichinohe et al., 2009). Thomas et al. (2009) demonstrated that, the innate immune response activation by NLRP3 inflammasomes is essential for animal protection. However, our understanding of the mechanisms of inflammasome antiviral defense remains limited (Anand et al., 2011).

Some viruses were shown to post-transcriptionally regulate inflammasome expression to benefit self-replication and propagation (Kieff and Rickinson, 2007; Rickinson and Kieff, 2007). For example, miRNA suppression of inflammasomes was shown in Epstein Barr Virus (EBV) infected cells (Kieff and Rickinson, 2007; Rickinson and Kieff, 2007). It appears that, EBV can avert NLRP3 inflammasome activation by expressing miRNAs encoded by three *BHRF1*-regions and 40 *BART*-regions of the viral genome (Albanese et al., 2016; Tagawa et al., 2016; Farrell, 2018). Additionally, two miRNAs encoded by EBV, *miR-BART11-5p* and *miR-BART15*, were identified by Haneklaus et al. (2012), which could bind to the 3′-UTR of *NLRP3*, the same site targeted by *miR-223*, and inhibit the inflammasome. It remains to be determined whether these viral miRNA could be used as therapeutic targets.

#### miRNA Regulation of Inflammasome in Autoimmune Diseases

Autoimmune diseases are often the result of a dysregulated immune response, characterized by inflammation and organ damage (Chang, 2013; Yang and Chiang, 2015). Chronic inflammation is frequently identified as a predisposing factor for an autoimmune reaction (Yang and Chiang, 2015). Multiple mechanisms were suggested to explain prolonged inflammation leading to autoimmunity; where failure to control inflammasome activation was recently identified in some autoimmune conditions (Yang and Chiang, 2015). It has been established that in addition to inflammation, an increased secretion of IL-1β and IL-18, can stimulate proliferation and organ distribution of the effector T cells, which can cause tissue damage (Oyanguren-Desez et al., 2011; Celhar et al., 2012). Therefore, targeting the inflammasome could be suggested to restore control over the inflammatory and immune response. Therapeutic potentials of several *NLRP3* targeting miRNAs were investigated in autoimmune diseases such as inflammatory bowel diseases (IBDs) (Neudecker et al., 2017), RA (Xie Z. et al., 2018), type 1 diabetes (T1D) (Yang and Chiang, 2015), type 2 diabetes (T2D) (Yang and Chiang, 2015), and systemic lupus erythematosus (SLE) (Zhu et al., 2012).

##### Inflammatory bowel diseases (IBDs)

Inflammatory bowel diseases are characterized by chronic inflammation of the intestine and comprise two disorders Crohn’s disease and ulcerative colitis. It is believed that the pathogenesis of IBDs is associated with dysregulation of innate and adaptive immune responses, triggered by microbial antigens. This could result in chronic inflammation of the digestive tract and damage to the intestinal mucosa (Fiocchi, 1998). The role of the inflammasome in intestinal inflammation is controversial. Zaki et al. (2010) reported that, NLRP3 induced production of IL-18 in intestinal epithelial cells can be protective, and contributes to epithelium integrity in experimental colitis. In contrast, Seo et al. (2015) have demonstrated the role of inflammasome in exacerbation of an intestinal pathology. The damaging effect of the inflammasome was also confirmed by Shouval et al. (2016), who identified that IL-1β inhibition improves the course of IBDs. It appears that increased IL-1β levels and tissue damage in IBDs are linked to NLRP3 activation in myeloid leukocytes infiltrating the gut tissue (Neudecker et al., 2017). The role of the inflammasome in IBDs pathogenesis was also confirmed by using a *miR-223* deficient animal model of colitis (Neudecker et al., 2017). *miR-223* deficient mice develop experimental colitis manifesting with colonic ulceration, inflammatory leukocyte infiltration and tissue injury which resembles closely IBDs (Neudecker et al., 2017). Tissue injury in these mice was linked to an enhanced NLRP3 expression and elevated IL-1β (Neudecker et al., 2017). Treatment of animals with *miR-223* mimetics alleviated symptoms of the colitis which coincided with reduced *NLRP3* RNA and IL-1β levels (Neudecker et al., 2017). This data presents *miR-223* as a novel biomarker and therapeutic target in subsets of IBDs and colitis (Polytarchou et al., 2015).

##### Rheumatoid arthritis (RA)

Rheumatoid arthritis is a chronic, systemic inflammatory disease affecting joints as well as skin, eyes, lungs, heart, and blood vessels (Scott et al., 2010). It was suggested that RA pathogenesis is related to activation of the NLRP3/IL-1β axis, where inflammasome activation was linked to worsening symptoms of the disease (Xie Q. et al., 2018). It was shown that activation of NLRP3 leads to an abundant expression of IL-1β (Guo et al., 2018), which can trigger T helper type 17 (Th17) cell differentiations and osteoclasts activation in RA (Dayer, 2003; McInnes and Schett, 2011; Zhang et al., 2015b). Th17 cells play a central role in RA pathogenesis, by maintaining chronic inflammation, recruiting neutrophils and promoting joint degradation (Cai et al., 2001; Shahrara et al., 2009; Leipe et al., 2010). Recently, an indirect effect of *miR-33* on NLRP3 activation was demonstrated in RA (Xie Q. et al., 2018), which could be explained by miRNA controlled dysregulation of mitochondrial function (Schroder et al., 2010; Zhou et al., 2011; Miao et al., 2014; Ouimet et al., 2015). Xie Q. et al. (2018) suggested that *miR-33* increases mitochondrial oxygen consumption and accumulation of reactive oxygen species which upregulates expression of NLRP3 and PCA1 in RA. Also, both *miR*-*33* expression and NLRP3 inflammasome activity were found to be higher in RA monocytes as compared to controls (Xie Q. et al., 2018). These findings indicate that *miR*-*33* could play an indirect role in pathogenesis of RA through NLRP3 inflammasome activation. Additional studies will provide more insight into the miRNA regulation of NLRP3 in RA and its therapeutic and prognostic implications.

##### Type 1 diabetes (T1D)

Type 1 diabetes is caused by autoimmune targeted elimination of pancreatic β cells islet (Kloppel et al., 1985). It was shown that TLRs play an essential role in the pathogenesis of T1D (Xie Z. et al., 2018). Upregulated expression of TLR4 as well as increased activity of the downstream targets was demonstrated in monocytes from T1D (Devaraj et al., 2008). Increased expression of activated TLRs was explained as a reaction to a high levels of circulating ligands in TID (Devaraj et al., 2009). Also, epigenetic regulation was associated with an aberrant TLR signaling and an increased IL-1β expression in T1D (Grishman et al., 2012). Several miRNAs were found altered in pre-TID patients, where levels of nine miRNAs (*miR-146a*, *miR-561*, and *miR-548a-3p*, *miR-184*, and *miR-200a*) were decreased, and two miRNAs (*miR-30c* and *miR-487a*) were increased (Grieco et al., 2018). Supporting these results was data published by Wang G. et al. (2018) demonstrating lower levels of *miR-150*, *miR-146a*, and *miR-424* compared to controls. One of the most consistent findings was the decreased *miR-146a* levels in T1D. It appears that *miR-146a* deficiency could play role in T1D exacerbation and increased IL-1β and IL-18 expression (Bhatt et al., 2016). Increased IL-1β levels could indicate inflammasome activation in T1D, although the role of inflammasome in the disease pathogenesis remains largely unknown.

##### Type 2 diabetes (T2D)

Circulating autoantibodies to β cells, self-reactive T cells and the glucose-lowering efficacy of some immunomodulatory therapies are suggestive of the autoimmune nature of the T2D (Itariu and Stulnig, 2014). Interestingly, a role for miRNA regulation of gene expression was demonstrated in T2D, where Balasubramanyam et al. (2011) have shown reduced *miR-146a* which was associated with increased *NF-κB*, *TNF-α* and *IL-6* mRNA levels. It is the same miRNA, which was found implicated to pathogenesis of T1D (Xie Z. et al., 2018), indicating potential similarities in the pathogenesis of both diseases. Recently *in vivo* studies demonstrated that *miR-146a* deficiency could increase expression of M1 and suppress expression of M2 markers in macrophages collected from patients with diabetes (Bhatt et al., 2016). Macrophage polarization occurs in the presence of IFNγ (M1) or IL-4 (M2) (Nathan et al., 1983; Stein et al., 1992) and is linked to pro-inflammatory and anti-inflammatory activities, respectively. M1 macrophages were shown to support inflammation by producing pro-inflammatory cytokines, including the inflammasome product IL-1β (Bhatt et al., 2016). Therefore, a link could be suggested between low *miR-146a* levels and inflammasome activation in M1 cells. More investigation is required to identify the connection between *miR-146a* and inflammasome activation and the role of this in T2D pathogenesis.

##### Systemic lupus erythematosus (SLE)

Systemic lupus erythematosus is an autoimmune disease caused by the loss of immune tolerance to ubiquitous autoantigens (Tsokos, 2011). Inflammation plays essential role in SLE pathogenesis (Yang et al., 2014; Rose and Dorner, 2017), where high levels of circulating proinflammatory cytokines are commonly detected (Yao et al., 2016; Mende et al., 2018). Inflammasome activation is proposed as one of the mechanisms underlying increased proinflammatory cytokine level in SLE (Kahlenberg and Kaplan, 2014). This assumption is supported by a report where IL-1β deficient mice were found to be resistant to experimental SLE (Voronov et al., 2006). Also, an increased expression of NLRP3 and AC1 have been reported in SLE nephritis biopsies (Kahlenberg et al., 2011). Kahlenberg and Kaplan (2014) have shown that SLE macrophages are highly reactive to innate immune stimuli, often leading to inflammasome activation. Therefore, targeting inflammasome activity could be a novel approach for SLE treatment. The expression of several miRNAs targeting the inflammasome and its products were found differentially expressed in SLE. For example, Wang et al. (2012) have demonstrated high levels of circulating *miR-223*, which was shown to inhibit *NLRP3*, in SLE. Also, reduced levels of circulating *miR-146a*, which regulates priming of TLRs, was found in SLE plasma (Wang et al., 2012). Interestingly, expression of *miR-23b*, which indirectly inhibits IL-1β responses, was shown to be downregulated in inflammatory lesions of SLE patients and animal model (Zhu et al., 2012). More studies are required to determine the role of miRNAs in pathogenesis of SLE and their therapeutic potential.

#### miRNA Regulation of Inflammasome in Neurodegenerative Disorders

Inflammasome products, IL-1β and IL-18, were shown to be essential for the health and functional competence of the nervous system (McAfoose and Baune, 2009; Dinarello et al., 2012). NLRP3 expression was demonstrated in microglia and astrocytes, which could explain the constitutive level of these cytokines in the brain (McAfoose and Baune, 2009; Dinarello et al., 2012; Savage et al., 2012; Minkiewicz et al., 2013; Cho et al., 2014; Lu M. et al., 2014). Interestingly, higher than normal levels of IL-1β and IL-18 were found in several neurodegenerative disorders, suggesting that over-activation of inflammasomes may play a role in pathogenesis of these diseases (Cho et al., 2014; Lu M. et al., 2014; Denes et al., 2015; Mamik and Power, 2017; Song et al., 2017). The significance of miRNA in the regulation of inflammasome activation in the pathogenesis of neurodegenerative diseases remains largely unknown. However, the role of an aberrant miRNA in regulation of *NLRP3* expression was previously demonstrated in Parkinson’s disease (PD).

Parkinson’s disease is a neurodegenerative disease which is characterized by progressive loss of dopaminergic neurons in substantia nigra compacta (Gasser, 2009). It is believed that accumulation of α-Syn fibrillary aggregates in the brain, most notably in the nigral dopaminergic neurons, induces the neuroinflammation (Eriksen et al., 2003). According to Zhou Y. et al. (2016), α-Syn can activate NLRP3 inflammasomes in microglia leading to an increased production of IL-1β. The authors also demonstrated that, *miR-7* and *miR-30e* analogs can inhibit NLRP3 inflammasome mediated neuroinflammation in the brain and protect dopaminergic neurons (Zhou Y. et al., 2016). It appears that the anti-inflammatory effects of *miR-7* and *miR-30e* are associated with their targeting of *NLRP3* mRNA in microglial cells. Interestingly, decreased *miR-7* and *miR-30e* expression was demonstrated in PD, which could lead to the loss of the regulatory control of α-Syn induced NLRP3 activation (Li D. et al., 2018).

#### miRNA Regulation of the Inflammasome in Cardiovascular Diseases (CVDs)

The physiological significance of inflammation is confirmed as it facilitates elimination of destructive stimuli and pathogens. However, aberrant inflammatory responses could cause tissue damage, tissue fibrosis and chronic diseases (Liu D. et al., 2018). Inflammation is recognized as a major risk factor for CVDs (Zhou et al., 2018), where chronic inflammasome activation was shown to contribute to the pathogenesis of atherosclerosis, ischemic and non-ischemic heart diseases (Zhou et al., 2018). Therefore, regulation of inflammasome activity using miRNA could be used for treatment and prevention of CVDs. Currently, strong evidence for the role of NLRP3 activation has been demonstrated in pathogenesis atherosclerosis.

Atherosclerosis is a form of CVD characterized by narrowing of the blood vessel lumen due to plaque formation, continuous dyslipidemia and inflammation (Ross, 1993). Chronic inflammation is commonly found in and around the atherosclerotic plaques which has an adverse effect on the arterial wall structure and function (Bernhagen et al., 2007). It is believed that atherogenic lipid mediators, involved in the formation of chronic inflammation in atherosclerotic plaque (Chen et al., 2006), can trigger peripheral blood monocytes migration and differentiation into macrophages within the intima of the arterial wall (Chen et al., 2006). T cells were also detected in atherosclerotic lesions (Kleemann et al., 2008), where, together with activated macrophages, they were shown to secrete proinflammatory mediators such as interferons, interleukins, and proteases (Østerud and Bjørklid, 2003; Shashkin et al., 2005; Tabas, 2005; Chen et al., 2006). IL-1β expression was identified in the early phase of atherosclerotic plaque formation and this stimulates secretion of additional cytokines and chemokines (Kleemann et al., 2008). Therefore, inflammasome activation in macrophages and T cell within the atherosclerotic lesion contributes to the pathogenesis of chronic inflammation.

*miR-22*, a miRNA inhibiting *NLRP3*, is decreased in peripheral blood mononuclear cells from coronary atherosclerosis (Chen B. et al., 2016), suggesting that upregulation of this miRNA could have therapeutic potential in CVD. Supporting this assumption, Huang W.Q. et al. (2017) investigated the effect of *miR-22* on the NLRP3 inflammasome and endothelial cell damage in an *in vivo* model of coronary heart disease. The authors demonstrated that *miR-22* mimics could decrease the release of inflammatory cytokines such as IL-1β and IL-18 by suppressing *NLRP3* expression in monocytes and macrophages (Huang W.Q. et al., 2017). Two additional miRNAs, *miR-9* and *mir-30e-5p* were found to indirectly affect inflammasome activation in atherosclerosis (Wang Y. et al., 2017; Li P. et al., 2018). It appears that *miR-9* could indirectly suppress inflammasome activation by targeting an atherogenic lipid mediator, oxidized low-density lipoprotein (oxLDL), in atherosclerosis (Liu W. et al., 2014). In another report, Wang Y. et al. (2017) reported that *miR-9* inhibits NLRP3 inflammasome activation induced by oxLDL in human THP-1 derived macrophages and peripheral blood monocytes in an *in vitro* atherosclerosis model. *miR-9* targets *Janus kinase 1* (*JAK1*) pathway (Wang Y. et al., 2017) inhibiting expression of NF-κB p65 which is required for the first step of NLRP3 inflammasome activation (Wang Y. et al., 2017). In addition, *miR-30c-5p* was linked to an indirect regulation of *NLRP3* expression in atherosclerosis (Li P. et al., 2018). Li P. et al. (2018) reported that *miR-30c-5p* protects human aortic endothelial cells (HAECs) from the oxLDL insult by targeting *FOXO3*. The authors showed that *miR-30c-5p* can suppress *FOXO3* expression and, consequently, decrease levels of NLRP3, AC1, IL-18 and IL-1β in HAECs (Li P. et al., 2018). As evidence emerges supporting the role of NLRP3 in the pathogenesis of atherosclerosis, targeting the inflammasome becomes an attractive therapeutic approach, where miRNAs could be suitable novel tools.

#### miRNA in Regulation of Inflammasome in Cancer

The role of the inflammasome in tumorigenesis remains controversial. Some reports indicate that NLRP3 inflammasome activation and IL-18 signaling protect against colorectal cancer (Karki et al., 2017), whereas progression of breast cancer, fibrosarcoma, gastric carcinoma, and lung metastasis were shown to be supported by the inflammasome (Okamoto et al., 2010; Kolb et al., 2014). Inflammasome regulation is complex, where multiple factors are implicated, making identification of the key regulatory elements challenging. As the inflammasome involvement in pathogenesis of some malignancies becomes more evident, understanding the regulatory mechanisms could lead to the discovery of novel therapeutic targets for cancer treatment.

##### Hepatocellular carcinoma (HCC)

Hepatocellular carcinoma (HCC) is a frequent sequelae of hepatitis B and hepatitis C viral infection (Perz et al., 2006). It is understood that these viruses activate NLRP3 inflammasomes causing hepatocyte pyroptosis, apoptosis and fibrosis (Kofahi et al., 2016). However, HCC tissue analysis failed to detect inflammasome activation; in fact, it was found to be significantly down-regulated when compared to the adjacent normal tissue (Zhu et al., 2011; Wei et al., 2014). To explain this inconsistency, Wei et al. (2014) suggested that *NLRP3* expression is dynamic changing during the progression of HCC. It appears that NLRP3 expression was increased in liver cells at the early stages of transformation, while inflammasome levels were decreased in malignant cells when compared to adjacent normal tissue (Wei et al., 2014). Interestingly, levels of *miR-223*, a negative regulator of *NLRP3*, were found to be increased in Hep3B cells derived from HCC (Wan et al., 2018). Increased *miR-223* was shown to coincide with tumor growth, suggesting a role in post-transcriptional mechanisms in malignant progression. In addition to *NLRP3*, *miR-223* was shown to target *erythrocyte membrane protein band 4.1 like 3* (*EPB41L3*) and *FOXO1* (Li and Rana, 2014; Kim et al., 2017). FOXO1 transcription factor binds to the thioredoxin-interacting protein (TXNIP) and regulates genes involved in cell death as well as the oxidative stress responses (Kim et al., 2017). TXNIP interacts with the NLRP3 inflammasome and activates AC1 in murine β-cells (Zhou et al., 2010). In addition, *miR-223* appears to be released systemically, where the level of this miRNA in the plasma was significantly lower in HCC cases (Giray et al., 2014). In addition to *miR-223*, decreased circulating *miR-30e*, which also targets *NLRP3*, was found in HCC cases (Bhattacharya et al., 2016). Therefore, it could be suggested that analysis of serum levels of *miR-223* and *miR-30e* could be used for diagnosis of HCC as well as an indicator of the efficacy of anticancer therapeutics.

##### Colorectal cancer (CRC)

Data on the role of NLRP3 in colorectal cancer (CRC) pathogenesis is inconsistent, where some evidence suggests a pro-tumorigenic role for the inflammasome, while others identified that the inflammasomes protects against tumor (Allen et al., 2010; Huber et al., 2012; Guo et al., 2014; Wang et al., 2016). Inflammasome expression analysis also demonstrated contradicting results where Wang et al. (2016) reported high NLRP3 in mesenchymal-like colon cancer cells, while Allen et al. (2010) demonstrated decreased inflammasome expression in colitis-associated cancer. Inflammasome contribution to tumorigenesis varies depending on the target cell type in the intestinal tissue (Lissner and Siegmund, 2011). According to Lissner and Siegmund (2011), inflammasome activation is required to maintain integrity of the epithelium. However, aggravated activation of the inflammasome stimulates intestinal inflammation, which could have a detrimental effect on epithelium permeability and increase its leakage (Lissner and Siegmund, 2011). It was identified that damage to the intestinal epithelium could trigger NLRP3 activation and secretion of IL-18, a proinflammatory cytokine (Huber et al., 2012). Subsequently, it was shown that IL-18 could reduce the expression of IL-22 binding protein (IL-22BP) and increase levels of IL-22 (Huber et al., 2012). Although IL-22 is protective against malignancies, aberrant over expression of IL-22 could trigger gut epithelial cell transformation and CRC development (Huber et al., 2012). Therefore, it is believed that IL-18, a NLRP3 product, has a promoting role in CRC development (Huber et al., 2012).

Targeting the inflammasome was suggested as a potential approach for treatment of CRC (Guo et al., 2014). NLRP3 expression was shown to be regulated by multiple miRNAs in various diseases (Haneklaus et al., 2012; Feng et al., 2018; Wan et al., 2018; Xie Q. et al., 2018). However, the role of miRNAs in cancer pathogenesis is not straight forward. There are inconsistent results regarding the expression status of *miR-223*, a known regulator of NLRP3 expression, in CRC cell lines and primary tumors. In a clinical study, the expression of *miR-223* was found to be significantly higher in stage III/IV patients (Ding J. et al., 2018). However, levels of *miR-223* vary significantly in colon tumor derived cell lines (Ding J. et al., 2018). Wu et al. (2012) reported reduced expression of *miR-223* in a HCT116, a CRC cell line. In contrast, several research groups demonstrated up-regulation of *miR-223* in CRC cell lines and primary tissues (Wang F. et al., 2017; Ju et al., 2018; Wei et al., 2018). Similar to these results, Ju et al. (2018) demonstrated up-regulation of *miR-223* in SW620, a CRC cell line. It was identified that high expression of *miR-223* suppresses *FoxO3a* and enhances cancer cell proliferation (Ju et al., 2018). It appears that the protumorigenic effect of Foxo3a is via NF-κB activation, which is essential for upregulation of the inflammasome linked proinflammatory signaling pathways (Thompson et al., 2015).

Unlike *miR-223*, data on miR-22 expression status in CRC consistently demonstrates that *miR-22* expression is significantly lower in CRC tissues and cell lines (Zhang et al., 2012, 2015a; Li B. et al., 2013; Xia et al., 2017; Liu Y. et al., 2018). Also, absence of *miR-22* was shown to positively correlate with increased cancer cell proliferation, migration, invasion, and metastasis (Zhang et al., 2012, 2015a; Li B. et al., 2013; Xia et al., 2017; Liu Y. et al., 2018). Multiple genes were identified as targets for *miR-22* including TIAM1 (Li B. et al., 2013), *BTG1* (Zhang et al., 2015a), *HuR* (Liu Y. et al., 2018), and *SP-1* (Xia et al., 2017). Among these genes, only *SP-1* gene expression was linked to inflammasome regulation (Hofmann et al., 2015). According to Hofmann et al. (2015), Sp-1 protein could contribute to NLRP3 inflammasome activation in monocytes in chronic recurrent multifocal osteomyelitis. However, the role of Sp-1 in activation of the NLRP3 inflammasome in CRC tumor tissues and monocytes remains largely unknown. Recent finding revealed that, in addition to *miR-22*, another negative regulator of NLRP3, *miR-30e*, is absent in CRC tumors as compared to normal colon tissues (Laudato et al., 2017). However, the role of *miR-30e* in CRC pathogenesis remains unknown.

##### Gastric cancer (GC)

It was shown that NLRP3 inflammasome activation promotes gastric cancer (GC) cells proliferation (Li S. et al., 2018). Over expression of *miR-223* supports GC invasion and metastasis in primary GC tumors (Haneklaus et al., 2012). Additionally, Li S. et al. (2018) reported that increased NLRP3 expression in GC tumors and macrophages negatively correlates with *miR-22* expression. The authors also demonstrated that constitutive expression of *miR-22* dramatically decreases *NLRP3* mRNA expression and IL-1β secretion in macrophages (Li S. et al., 2018). Therefore, the effect of targeting *NLRP3* expression with miRNAs in tumors and immune cells may vary depending on tumor and/or cell type.

##### Oral squamous cell carcinoma (OSCC)

High *NLRP3* expression was found in oral squamous cell carcinoma (OSCC) cells and tissues (Wang H. et al., 2018). A role for *NLRP3* supporting OSCC proliferation and growth was demonstrated in several reports. Wang G. et al. (2018) demonstrated a positive correlation between *NLRP3* expression and tumor size, lymph node status and IL-1β expression in OSCC tissue specimens and *in vivo* models of OSCC. Also, the authors showed that, silencing of *NLRP3* in OSCC cell lines reduced cell proliferation, migration, and invasion *in vitro* (Wang H. et al., 2018). Additionally, high expression of the NLRP3 inflammasome mediates chemoresistance in OSCC (Feng et al., 2018). Therefore, downregulation of *NLRP3* could have a therapeutic potential in OSCC.

Surprisingly, high expression of *miR-223*, which targets *NLRP3*, was found in primary OSCC tissue (Manikandan et al., 2016). *In silico* analysis identified a *Ras Homolog Family Member B* (*RHOB*) as a potential target for *miR-223* in OSCC (Manikandan et al., 2016). It appears that *miR-223* could indirectly suppress NLRP3 and TLR4/NF-κB signaling via RHOB (Yan et al., 2019). These data provide a novel potential target for OSCC treatment, where *miR-223* inhibition of NLRP3 could be attained through RHOB.

Overexpression of *miR-22* in OSCC was shown to reduce NLRP3 activation and decrease OSCC malignancy (Feng et al., 2018). *miR-22* levels were shown to be inversely correlated with NLRP3 expression and *miR-22* levels were significantly lower in OSCC compared to adjacent non-cancerous tissue (Feng et al., 2018). The inhibitory effect of *miR-22* on OSCC migration was confirmed using a lentiviral expression system. As expected an inhibitor of *miR-22* promoted OSCC spread (Feng et al., 2018). The 3′-UTR of the *NLRP3* gene was identified as a *miR-22* target site (Feng et al., 2018). It appears that NLRP3 promotes OSCC growth and tumor spread, which makes *miR-22* a potential therapeutic target for cancer treatment. Two miRNAs, *miR-223* and *miR-22*, were identified as inhibiting the inflammasome and, subsequently, suppressing tumor growth. Therefore, the anti-tumor effect of these molecules in OSCC warrants further investigation.

##### Cervical cancer (CC)

Human papillomavirus (HPV) infection and persistent chronic inflammation were identified as fundamental for the pathogenesis of cervical cancer (CC) (de Castro-Sobrinho et al., 2016; Kriek et al., 2016). HPV can cause chronic inflammation by inducing TLR4 expression and impairing the TLR4-NF-κB pathway (Wang et al., 2014; He A. et al., 2016).

Wu et al. (2012) reported reduced expression of *miR-223*, which targets *NLRP3*, in the CC cell line HeLa. The authors also demonstrated that over-expression of *miR-223* inhibits tumor cell proliferation by targeting *FOXO1* (Wu et al., 2012). In addition, another direct post-transcriptional regulator of *NLRP3*, miR-22, was found to be down-regulated in CC cell lines and tissues (Xin et al., 2016; Wongjampa et al., 2018). Furthermore, Wongjampa et al. (2018) reported an inverse correlation between histone deacetylase 6 (HDAC6) and *miR-22*. It was previously shown that HDAC6 directly binds to NLRP3 via its ubiquitin-binding domain to regulate NLRP3 inflammasome expression (Hwang et al., 2015). As NLRP3 plays a role in the pathogenesis of HPV induced chronic inflammation, *miR-223* and *miR-22*, both of which regulate inflammasome activation, could be potential therapeutic tools for the treatment of CC.

##### Glioblastoma (GBM)

High NLRP3 inflammasome activation and high levels of inflammasome products are found in malignant glioblastoma (GBM) (Basu et al., 2004; Tarassishin et al., 2014). Increased IL-1β, a major NLRP3 inflammasome product, was linked to the release of VEGF and MMPs, angiogenic factors, in human astrocytes and GBM cells (Suh et al., 2013). Therefore, it could be suggested that inflammasome activation favors GBM growth and spread.

Several miRNAs were shown to regulate inflammasome expression, where decreased miRNA levels could promote GBM growth and invasion. Ding Q. et al. (2018) demonstrated that *miR-223*, which is effective at reducing NLRP3 inflammasome levels in several tumors (Wu et al., 2012), was decreased in GBM tissues (Ding Q. et al., 2018). However, a conflicting report from Cheng et al. (2017) indicated that *miR-223* is overexpressed in GBM cell lines. Similar findings were also reported in GBM stem like cells and GBM tissues (Huang B.S. et al., 2017). Similarly there are conflicting data regarding *miR-223* targets and phenotypic impacts. A *miR-223-3p* mimic inhibited tumor cell proliferation and migration, effects that were due to a reduction in proinflammatory cytokines IL-1β and IL-18 in GBM cell lines (Ding Q. et al., 2018). Also, nuclear factor I-A (NFIA) was a target of *miR-223* in GBM cell lines and was found to decrease tumorigenesis in the CNS (Glasgow et al., 2013). The pro-tumorigenic effect of *miR-223* was linked to suppression of the tumor suppressor *paired box 6* (*PAX6*) (Cheng et al., 2017). By targeting *PAX6*, *miR-223* could promote GBM stem cell chemotherapy resistance (Huang B.S. et al., 2017). The mechanism underlying the diverse effects of *miR-223* on GBM growth and metastasis remains largely unknown. However, it could be suggested that the stage of tumorigenesis plays a role in the effect of *miR-223* in GBM.

Levels of *miR-22 and miR-30e*, two post-transcriptional regulators of *NLRP3*, are low in GBM tissues (Li W.B. et al., 2013; Chakrabarti et al., 2016; Chen H. et al., 2016). In addition to targeting *NLRP3*, *miR-22* can also directly target the 3′-UTRs of *SIRT1* (Li W.B. et al., 2013), and *miR-22* mimics decrease the expression of SIRT1 protein in GBM cell lines (Li W.B. et al., 2013). Interestingly, several studies have demonstrated that SIRT1 can suppress NLRP3 (Ma et al., 2015; Jiang et al., 2016; Zhou C.C. et al., 2016). It could be proposed that the decreased levels of *miR-22* could fail to control *NLRP3* expression, which could enable GMB tumorigenesis.

## Future Aspects for Clinical Approaches

The role of the NLRP3 inflammasome in the pathogenesis of several diseases was demonstrated, including CAPS, autoimmune disorders and cancers (Aganna et al., 2002; Martinon et al., 2006; Masters et al., 2009; Bauer et al., 2010; Wen et al., 2011). An increased IL-1β level, commonly found in these diseases, is a strong indicator of NLRP3 inflammasome activation. Also, the body of evidence suggests that IL-1β plays a central role in disease pathogenesis. Therefore, targeting IL-1β, a NLRP3 inflammasome product, appears to be a rational therapeutic approach. The efficacy of anti-IL-1β therapy was demonstrated in CAPS, where both the symptoms and severity of the disease were alleviated using either an IL-1β receptor antagonist or anti-IL-1β antibodies (Hoffman et al., 2008; Dinarello, 2009; Lachmann et al., 2009). A similar approach targeting IL-1β was successfully applied to treat NLRP3 inflammasome associated autoimmune diseases and cancer (Larsen et al., 2007; Lust et al., 2009). These data provide compelling evidence for the NLRP3 inflammasome as a potential therapeutic target for treatment of the diseases associated with an elevated level of IL-1β. In this respect, miRNAs have therapeutic potentials as they could target *NLRP3* preventing its expression and, consequently, averting IL-1β production.

miRNA based replacement and silencing therapeutic approaches were tested in several preclinical and clinical studies (Li and Rana, 2014). miRNAs and miRNA-targeting oligonucleotides approaches (mimic and/or anti-miR technologies) appear to be more effective when compared to small-molecule drugs due to their ability to effect concurrently multiple gene targets (Li and Rana, 2014). Anti-*miR-122* oligonucleotide, Miravirsen, was the first miRNA-based therapeutic used to treat hepatitis c infection (Lindow and Kauppinen, 2012; van der Ree et al., 2016). Currently Miravirsen is in a phase II clinical trial (van der Ree et al., 2016). Several phase I clinical trials and pre-clinical studies using miRNA-targeting oligonucleotide technologies targeted to *Let-7*, *miR-10b*, *miR-21*, *miR-34*, *miR-155*, *miR-221*, *and others*, have demonstrated positive results (Moles, 2017). miRNA-targeting oligonucleotides are designed to bind to their targeted miRNA (Li and Rana, 2014). miRNAs generally target more than one gene in the same signaling pathway (Li Z. et al., 2011; Li and Rana, 2014). This feature of miRNAs makes them valuable as therapeutic candidates (Li and Rana, 2014).

However, there are still multiple obstacles to overcome, including target specificity and the potential toxicity of miRNA-targeting oligonucleotides (Merhautova et al., 2016). First, the limited specificity, anti-miRs generally target nucleotide sequences on miRNAs which can be present on multiple miRNAs within the same family (Hogan et al., 2014). Chemical modifications of anti-miRs have been suggested to improve their specificity (Hogan et al., 2014). Second, when administered without a carrier molecule, their effect may be limited and they can be cleared by the liver and kidney (Bennett and Swayze, 2010). Third, anti-miRs can be sensed and eliminated by receptors of the innate and adaptive immune responses (Diebold et al., 2004; Heil et al., 2004). To overcome this limitation, tissue specific antibody coated chemically engineered polymer-based nanoparticles and carrier proteins have been developed to improve the specificity and efficacy of delivery. For example, the therapeutic efficiency of *miR-223* was improved by using nanoparticle lipid emulsions as a delivery method, in animal model of colitis (Neudecker et al., 2017). These exciting results demonstrate great potential for miRNA-based treatments of diseases linked to NLRP3 dysfunction.

Our understanding of the role of the inflammasome in disease pathogenesis is still limited and is hampering development of the miRNA targeting therapeutics against the inflammasome. However, exciting discoveries in fundamental and preclinical research in recent years have demonstrated great potential for miRNA targeting in the treatment of diseases linked to NLRP3 dysfunction.

## Author Contributions

GT and SK contributed to the conception and design of the study. ZG organized the database. SK wrote the first draft of the manuscript. GT, EM, ZG, AM, AR, and SK wrote sections of the manuscript. All authors contributed to manuscript revision, read and approved the submitted version.

## Conflict of Interest Statement

The authors declare that the research was conducted in the absence of any commercial or financial relationships that could be construed as a potential conflict of interest.

## References

[B1] Afonina I. S. Muller C. Martin S. J. Beyaert R. (2015). Proteolytic processing of interleukin-1 family cytokines: variations on a common theme. *Immunity* 42 991–1004. 10.1016/j.immuni.2015.06.003 26084020

[B2] Aganna E. Martinon F. Hawkins P. N. Ross J. B. Swan D. C. Booth D. R. (2002). Association of mutations in the NALP3/CIAS1/PYPAF1 gene with a broad phenotype including recurrent fever, cold sensitivity, sensorineural deafness, and AA amyloidosis. *Arthritis Rheum.* 46 2445–2452. 10.1002/art.10509 12355493

[B3] Agostini L. Martinon F. Burns K. McDermott M. F. Hawkins P. N. Tschopp J. (2004). NALP3 forms an IL-1beta-processing inflammasome with increased activity in Muckle-Wells autoinflammatory disorder. *Immunity* 20 319–325. 15030775 10.1016/s1074-7613(04)00046-9

[B4] Albanese M. Tagawa T. Bouvet M. Maliqi L. Lutter D. Hoser J. (2016). Epstein-Barr virus microRNAs reduce immune surveillance by virus-specific CD8+ T cells. *Proc. Natl. Acad. Sci. U.S.A.* 113 E6467–E6475. 10.1073/pnas.1605884113 27698133 PMC5081573

[B5] Alexanderála Pazár M. D. (2010). *The Expanded Role of the Inflammasome in Human Disease*. Available at: https://www.the-rheumatologist.org/article/the-expanded-role-of-the-inflammasome-in-human-disease/ (accessed August 01, 2010).

[B6] Allam R. Lawlor K. E. Yu E. C. Mildenhall A. L. Moujalled D. M. Lewis R. S. (2014). Mitochondrial apoptosis is dispensable for NLRP3 inflammasome activation but non-apoptotic caspase-8 is required for inflammasome priming. *EMBO Rep.* 15 982–990. 10.15252/embr.201438463 24990442 PMC4198042

[B7] Allen I. C. Scull M. A. Moore C. B. Holl E. K. McElvania-TeKippe E. Taxman D. J. (2009). The NLRP3 inflammasome mediates in vivo innate immunity to influenza A virus through recognition of viral RNA. *Immunity* 30 556–565. 10.1016/j.immuni.2009.02.005 19362020 PMC2803103

[B8] Allen I. C. TeKippe E. M. Woodford R.-M. T. Uronis J. M. Holl E. K. Rogers A. B. (2010). The NLRP3 inflammasome functions as a negative regulator of tumorigenesis during colitis-associated cancer. *J. Exp. Med.* 207 1045–1056. 10.1084/jem.20100050 20385749 PMC2867287

[B9] Allen I. C. Wilson J. E. Schneider M. Lich J. D. Roberts R. A. Arthur J. C. (2012). NLRP12 suppresses colon inflammation and tumorigenesis through the negative regulation of noncanonical NF-kappaB signaling. *Immunity* 36 742–754. 10.1016/j.immuni.2012.03.012 22503542 PMC3658309

[B10] Alnemri E. S. Livingston D. J. Nicholson D. W. Salvesen G. Thornberry N. A. Wong W. W. (1996). Human ICE/CED-3 protease nomenclature. *Cell* 87:171. 10.1016/s0092-8674(00)81334-3 8861900

[B11] Anand P. K. Malireddi R. K. Kanneganti T. D. (2011). Role of the nlrp3 inflammasome in microbial infection. *Front. Microbiol.* 2:12 10.3389/fmicb.2011.00012 PMC3109351 21687408

[B12] Anand P. K. Malireddi R. K. Lukens J. R. Vogel P. Bertin J. Lamkanfi M. (2012). NLRP6 negatively regulates innate immunity and host defence against bacterial pathogens. *Nature* 488 389–393. 10.1038/nature11250 22763455 PMC3422416

[B13] Androulidaki A. Iliopoulos D. Arranz A. Doxaki C. Schworer S. Zacharioudaki V. (2009). The kinase Akt1 controls macrophage response to lipopolysaccharide by regulating microRNAs. *Immunity* 31 220–231. 10.1016/j.immuni.2009.06.024 19699171 PMC2865583

[B14] Anzola A. Gonzalez R. Gamez-Belmonte R. Ocon B. Aranda C. J. Martinez-Moya P. (2018). miR-146a regulates the crosstalk between intestinal epithelial cells, microbial components and inflammatory stimuli. *Sci. Rep.* 8:17350. 10.1038/s41598-018-35338-y 30478292 PMC6255912

[B15] Arthur J. C. Lich J. D. Ye Z. Allen I. C. Gris D. Wilson J. E. (2010). Cutting edge: NLRP12 controls dendritic and myeloid cell migration to affect contact hypersensitivity. *J. Immunol.* 185 4515–4519. 10.4049/jimmunol.1002227 20861349 PMC3641837

[B16] Balasubramanyam M. Aravind S. Gokulakrishnan K. Prabu P. Sathishkumar C. Ranjani H. (2011). Impaired miR-146a expression links subclinical inflammation and insulin resistance in Type 2 diabetes. *Mol. Cell. Biochem.* 351 197–205. 10.1007/s11010-011-0727-3 21249428

[B17] Bartel D. P. (2004). MicroRNAs: genomics, biogenesis, mechanism, and function. *Cell* 116 281–297. 14744438 10.1016/s0092-8674(04)00045-5

[B18] Bartel D. P. (2009). MicroRNAs: target recognition and regulatory functions. *Cell* 136 215–233. 10.1016/j.cell.2009.01.002 19167326 PMC3794896

[B19] Basu A. Krady J. K. Levison S. W. (2004). Interleukin-1: a master regulator of neuroinflammation. *J. Neurosci. Res.* 78 151–156. 15378607 10.1002/jnr.20266

[B20] Bauer C. Duewell P. Mayer C. Lehr H. A. Fitzgerald K. A. Dauer M. (2010). Colitis induced in mice with dextran sulfate sodium (DSS) is mediated by the NLRP3 inflammasome. *Gut* 59 1192–1199. 10.1136/gut.2009.197822 20442201

[B21] Bauernfeind F. Rieger A. Schildberg F. A. Knolle P. A. Schmid-Burgk J. L. Hornung V. (2012). NLRP3 inflammasome activity is negatively controlled by miR-223. *J. Immunol.* 189 4175–4181. 10.4049/jimmunol.1201516 22984082

[B22] Bauernfeind F. G. Horvath G. Stutz A. Alnemri E. S. MacDonald K. Speert D. (2009). Cutting edge: NF-kappaB activating pattern recognition and cytokine receptors license NLRP3 inflammasome activation by regulating NLRP3 expression. *J. Immunol.* 183 787–791. 10.4049/jimmunol.0901363 19570822 PMC2824855

[B23] Bayarsaihan D. (2011). Epigenetic mechanisms in inflammation. *J. Dent. Res.* 90 9–17. 10.1177/0022034510378683 21178119 PMC3144097

[B24] Bennett C. F. Swayze E. E. (2010). RNA targeting therapeutics: molecular mechanisms of antisense oligonucleotides as a therapeutic platform. *Annu. Rev. Pharmacol. Toxicol.* 50 259–293. 10.1146/annurev.pharmtox.010909.105654 20055705

[B25] Berger S. L. (2007). The complex language of chromatin regulation during transcription. *Nature* 447 407–412. 17522673 10.1038/nature05915

[B26] Berger S. L. Kouzarides T. Shiekhattar R. Shilatifard A. (2009). An operational definition of epigenetics. *Genes Dev.* 23 781–783. 10.1101/gad.1787609 19339683 PMC3959995

[B27] Bernhagen J. Krohn R. Lue H. Gregory J. L. Zernecke A. Koenen R. R. (2007). MIF is a noncognate ligand of CXC chemokine receptors in inflammatory and atherogenic cell recruitment. *Nat. Med.* 13 587–596. 17435771 10.1038/nm1567

[B28] Bernot A. da Silva C. Petit J. L. Cruaud C. Caloustian C. Castet V. (1998). Non-founder mutations in the MEFV gene establish this gene as the cause of familial Mediterranean fever (FMF). *Hum. Mol. Genet.* 7 1317–1325. 9668175 10.1093/hmg/7.8.1317

[B29] Bhatt K. Lanting L. L. Jia Y. Yadav S. Reddy M. A. Magilnick N. (2016). Anti-inflammatory role of MicroRNA-146a in the pathogenesis of diabetic nephropathy. *J. Am. Soc. Nephrol.* 27 2277–2288. 10.1681/ASN.2015010111 26647423 PMC4978034

[B30] Bhattacharya S. Steele R. Shrivastava S. Chakraborty S. Di Bisceglie A. M. Ray R. B. (2016). Serum miR-30e and miR-223 as Novel Noninvasive Biomarkers for Hepatocellular Carcinoma. *Am. J. Pathol.* 186 242–247. 10.1016/j.ajpath.2015.10.003 26776075 PMC4729242

[B31] Bierne H. Hamon M. Cossart P. (2012). Epigenetics and bacterial infections. *Cold Spring Harb. Perspect. Med.* 2:a010272. 10.1101/cshperspect.a010272 23209181 PMC3543073

[B32] Boekhout A. H. Vincent A. D. Dalesio O. B. van den Bosch J. Foekema-Tons J. H. Adriaansz S. (2011). Management of hot flashes in patients who have breast cancer with venlafaxine and clonidine: a randomized, double-blind, placebo-controlled trial. *J. Clin. Oncol.* 29 3862–3868. 10.1200/JCO.2010.33.1298 21911720

[B33] Bonora M. Wieckowsk M. R. Chinopoulos C. Kepp O. Kroemer G. Galluzzi L. (2015). Molecular mechanisms of cell death: central implication of ATP synthase in mitochondrial permeability transition. *Oncogene* 34:1608. 10.1038/onc.2014.462 25790189

[B34] Bortolotti P. Faure E. Kipnis E. (2018). Inflammasomes in tissue damages and immune disorders after trauma. *Front. Immunol.* 9:1900. 10.3389/fimmu.2018.01900 30166988 PMC6105702

[B35] Boucher D. Monteleone M. Coll R. C. Chen K. W. Ross C. M. Teo J. L. (2018). Caspase-1 self-cleavage is an intrinsic mechanism to terminate inflammasome activity. *J. Exp. Med.* 215 827–840. 10.1084/jem.20172222 29432122 PMC5839769

[B36] Bourc’his D. Bestor T. H. (2004). Meiotic catastrophe and retrotransposon reactivation in male germ cells lacking Dnmt3L. *Nature* 431 96–99. 10.1038/nature02886 15318244

[B37] Bourc’his D. Xu G. L. Lin C. S. Bollman B. Bestor T. H. (2001). Dnmt3L and the establishment of maternal genomic imprints. *Science* 294 2536–2539. 10.1126/science.1065848 11719692

[B38] Boyden E. D. Dietrich W. F. (2006). Nalp1b controls mouse macrophage susceptibility to anthrax lethal toxin. *Nat. Genet.* 38 240–244. 10.1038/ng1724 16429160

[B39] Broz P. Newton K. Lamkanfi M. Mariathasan S. Dixit V. M. Monack D. M. (2010). Redundant roles for inflammasome receptors NLRP3 and NLRC4 in host defense against *Salmonella*. *J. Exp. Med.* 207 1745–1755. 10.1084/jem.20100257 20603313 PMC2916133

[B40] Burckstummer T. Baumann C. Bluml S. Dixit E. Durnberger G. Jahn H. (2009). An orthogonal proteomic-genomic screen identifies AIM2 as a cytoplasmic DNA sensor for the inflammasome. *Nat. Immunol.* 10 266–272. 10.1038/ni.1702 19158679

[B41] Cai L. Yin J. P. Starovasnik M. A. Hogue D. A. Hillan K. J. Mort J. S. (2001). Pathways by which interleukin 17 induces articular cartilage breakdown in vitro and in vivo. *Cytokine* 16 10–21. 10.1006/cyto.2001.0939 11669582

[B42] Canna S. W. de Jesus A. A. Gouni S. Brooks S. R. Marrero B. Liu Y. (2014). An activating NLRC4 inflammasome mutation causes autoinflammation with recurrent macrophage activation syndrome. *Nat. Genet.* 46 1140–1146. 10.1038/ng.3089 25217959 PMC4177369

[B43] Carlsson F. Kim J. Dumitru C. Barck K. H. Carano R. A. Sun M. (2010). Host-detrimental role of Esx-1-mediated inflammasome activation in mycobacterial infection. *PLoS Pathog.* 6:e1000895. 10.1371/journal.ppat.1000895 20463815 PMC2865529

[B44] Celhar T. Magalhaes R. Fairhurst A. M. (2012). TLR7 and TLR9 in SLE: when sensing self goes wrong. *Immunol. Res.* 53 58–77. 10.1007/s12026-012-8270-1 22434514

[B45] Ceppi M. Pereira P. M. Dunand-Sauthier I. Barras E. Reith W. Santos M. A. (2009). MicroRNA-155 modulates the interleukin-1 signaling pathway in activated human monocyte-derived dendritic cells. *Proc. Natl. Acad. Sci. U.S.A.* 106 2735–2740. 10.1073/pnas.0811073106 19193853 PMC2650335

[B46] Chakrabarti M. Klionsky D. J. Ray S. K. (2016). miR-30e blocks autophagy and acts synergistically with proanthocyanidin for inhibition of AVEN and BIRC6 to increase apoptosis in glioblastoma stem cells and glioblastoma SNB19 cells. *PLoS One* 11:e0158537. 10.1371/journal.pone.0158537 27388765 PMC4936720

[B47] Chang C. (2013). The pathogenesis of neonatal autoimmune and autoinflammatory diseases: a comprehensive review. *J. Autoimmun.* 41 100–110. 10.1016/j.jaut.2012.12.010 23375846

[B48] Chen B. Luo L. Zhu W. Wei X. Li S. Huang Y. (2016). miR-22 contributes to the pathogenesis of patients with coronary artery disease by targeting MCP-1: an observational study. *Medicine* 95:e4418. 10.1097/MD.0000000000004418 27537567 PMC5370794

[B49] Chen H. Lu Q. Fei X. Shen L. Jiang D. Dai D. (2016). miR-22 inhibits the proliferation, motility, and invasion of human glioblastoma cells by directly targeting SIRT1. *Tumor Biol.* 37 6761–6768. 10.1007/s13277-015-4575-8 26662303

[B50] Chen I. Y. Ichinohe T. (2015). Response of host inflammasomes to viral infection. *Trends Microbiol.* 23 55–63. 10.1016/j.tim.2014.09.007 25456015

[B51] Chen J.-W. Chen Y.-H. Lin S.-J. (2006). Long-term exposure to oxidized low-density lipoprotein enhances tumor necrosis factor-α-stimulated endothelial adhesiveness of monocytes by activating superoxide generation and redox-sensitive pathways. *Free Radic. Biol. Med.* 40 817–826. 16520234 10.1016/j.freeradbiomed.2005.10.037

[B52] Chen X. M. O’Hara S. P. Nelson J. B. Splinter P. L. Small A. J. Tietz P. S. (2005). Multiple TLRs are expressed in human cholangiocytes and mediate host epithelial defense responses to *Cryptosporidium parvum* via activation of NF-kappaB. *J. Immunol.* 175 7447–7456. 16301652 10.4049/jimmunol.175.11.7447

[B53] Chen X. M. Splinter P. L. O’Hara S. P. LaRusso N. F. (2007). A cellular micro-RNA, let-7i, regulates Toll-like receptor 4 expression and contributes to cholangiocyte immune responses against *Cryptosporidium parvum* infection. *J. Biol. Chem.* 282 28929–28938. 10.1074/jbc.M702633200 17660297 PMC2194650

[B54] Cheng Q. Ma X. Cao H. Chen Z. Wan X. Chen R. (2017). Role of miR-223/paired box 6 signaling in temozolomide chemoresistance in glioblastoma multiforme cells. *Mol. Med. Rep.* 15 597–604. 10.3892/mmr.2016.6078 28035389 PMC5364831

[B55] Cho M.-H. Cho K. Kang H.-J. Jeon E.-Y. Kim H.-S. Kwon H.-J. (2014). Autophagy in microglia degrades extracellular β-amyloid fibrils and regulates the NLRP3 inflammasome. *Autophagy* 10 1761–1775. 10.4161/auto.29647 25126727 PMC4198361

[B56] Choubey D. (2012). Interferon-inducible Ifi200-family genes as modifiers of lupus susceptibility. *Immunol. Lett.* 147 10–17. 10.1016/j.imlet.2012.07.003 22841963 PMC3425670

[B57] Cirelli K. M. Gorfu G. Hassan M. A. Printz M. Crown D. Leppla S. H. (2014). Inflammasome sensor NLRP1 controls rat macrophage susceptibility to *Toxoplasma gondii*. *PLoS Pathog.* 10:e1003927. 10.1371/journal.ppat.1003927 24626226 PMC3953412

[B58] Cohen T. S. Boland M. L. Boland B. B. Takahashi V. Tovchigrechko A. Lee Y. (2018). *S. aureus* evades macrophage killing through NLRP3-dependent effects on mitochondrial trafficking. *Cell Rep.* 22 2431–2441. 10.1016/j.celrep.2018.02.027 29490278 PMC7160668

[B59] Coll R. C. O’Neill L. A. (2010). New insights into the regulation of signalling by toll-like receptors and nod-like receptors. *J. Innate Immun.* 2 406–421. 10.1159/000315469 20505309

[B60] Costa A. Gupta R. Signorino G. Malara A. Cardile F. Biondo C. (2012). Activation of the NLRP3 inflammasome by group B streptococci. *J. Immunol.* 188 1953–1960. 10.4049/jimmunol.1102543 22250086 PMC3273589

[B61] Craven R. R. Gao X. Allen I. C. Gris D. Bubeck Wardenburg J. McElvania-Tekippe E. (2009). *Staphylococcus aureus* alpha-hemolysin activates the NLRP3-inflammasome in human and mouse monocytic cells. *PLoS One* 4:e7446. 10.1371/journal.pone.0007446 19826485 PMC2758589

[B62] Cridland J. A. Curley E. Z. Wykes M. N. Schroder K. Sweet M. J. Roberts T. L. (2012). The mammalian PYHIN gene family: phylogeny, evolution and expression. *BMC Evol. Biol.* 12:140. 10.1186/1471-2148-12-140 22871040 PMC3458909

[B63] Davis B. K. Wen H. Ting J. P. (2011). The inflammasome NLRs in immunity, inflammation, and associated diseases. *Annu. Rev. Immunol.* 29 707–735. 10.1146/annurev-immunol-031210-101405 21219188 PMC4067317

[B64] Davis E. E. Zhang Q. Liu Q. Diplas B. H. Davey L. M. Hartley J. (2011). TTC21B contributes both causal and modifying alleles across the ciliopathy spectrum. *Nat. Genet.* 43 189–196. 10.1038/ng.756 21258341 PMC3071301

[B65] Dayer J. M. (2003). The pivotal role of interleukin-1 in the clinical manifestations of rheumatoid arthritis. *Rheumatology* 42(Suppl. 2), ii3–ii10. 12817089 10.1093/rheumatology/keg326

[B66] de Castro-Sobrinho J. M. Rabelo-Santos S. H. Fugueiredo-Alves R. R. Derchain S. Sarian L. O. Z. Pitta D. R. (2016). Bacterial vaginosis and inflammatory response showed association with severity of cervical neoplasia in HPV-positive women. *Diagn. Cytopathol.* 44 80–86. 10.1002/dc.23388 26644228

[B67] Delaloye J. Roger T. Steiner-Tardivel Q. G. Le Roy D. Knaup Reymond M. Akira S. (2009). Innate immune sensing of modified vaccinia virus Ankara (MVA) is mediated by TLR2-TLR6, MDA-5 and the NALP3 inflammasome. *PLoS Pathog.* 5:e1000480. 10.1371/journal.ppat.1000480 19543380 PMC2691956

[B68] Denes A. Coutts G. Lénárt N. Cruickshank S. M. Pelegrin P. Skinner J. (2015). AIM2 and NLRC4 inflammasomes contribute with ASC to acute brain injury independently of NLRP3. *Proc. Natl. Acad. Sci. U.S.A.* 112 4050–4055. 10.1073/pnas.1419090112 25775556 PMC4386342

[B69] Devaraj S. Dasu M. R. Park S. H. Jialal I. (2009). Increased levels of ligands of Toll-like receptors 2 and 4 in type 1 diabetes. *Diabetologia* 52 1665–1668. 10.1007/s00125-009-1394-8 19455302 PMC2709882

[B70] Devaraj S. Dasu M. R. Rockwood J. Winter W. Griffen S. C. Jialal I. (2008). Increased toll-like receptor (TLR) 2 and TLR4 expression in monocytes from patients with type 1 diabetes: further evidence of a proinflammatory state. *J. Clin. Endocrinol. Metab.* 93 578–583. 10.1210/jc.2007-2185 18029454 PMC2243229

[B71] Diebold S. S. Kaisho T. Hemmi H. Akira S. Reis e Sousa C. (2004). Innate antiviral responses by means of TLR7-mediated recognition of single-stranded RNA. *Science* 303 1529–1531. 10.1126/science.1093616 14976261

[B72] Dihlmann S. Tao S. Echterdiek F. Herpel E. Jansen L. Chang-Claude J. (2014). Lack of Absent in Melanoma 2 (AIM2) expression in tumor cells is closely associated with poor survival in colorectal cancer patients. *Int J. Cancer* 135 2387–2396. 10.1002/ijc.28891 24729378

[B73] Dinarello C. A. (2009). Immunological and inflammatory functions of the interleukin-1 family. *Annu. Rev. Immunol.* 27 519–550. 10.1146/annurev.immunol.021908.132612 19302047

[B74] Dinarello C. A. Simon A. van der Meer J. W. (2012). Treating inflammation by blocking interleukin-1 in a broad spectrum of diseases. *Nat. Rev. Drug Discov.* 11 633–652. 10.1038/nrd3800 22850787 PMC3644509

[B75] Ding J. Zhao Z. Song J. Luo B. Huang L. (2018). MiR-223 promotes the doxorubicin resistance of colorectal cancer cells via regulating epithelial-mesenchymal transition by targeting FBXW7. *Acta Biochim. Biophys. Sin.* 50 597–604. 10.1093/abbs/gmy040 29701752

[B76] Ding Q. Shen L. Nie X. Lu B. Pan X. Su Z. (2018). MiR-223-3p overexpression inhibits cell proliferation and migration by regulating inflammation-associated cytokines in glioblastomas. *Pathol. Res. Pract.* 214 1330–1339. 10.1016/j.prp.2018.05.012 30033329

[B77] Dorhoi A. Iannaccone M. Farinacci M. Fae K. C. Schreiber J. Moura-Alves P. (2013). MicroRNA-223 controls susceptibility to tuberculosis by regulating lung neutrophil recruitment. *J. Clin. Invest.* 123 4836–4848. 10.1172/JCI67604 24084739 PMC3809781

[B78] Dostert C. Petrilli V. Van Bruggen R. Steele C. Mossman B. T. Tschopp J. (2008). Innate immune activation through Nalp3 inflammasome sensing of asbestos and silica. *Science* 320 674–677. 10.1126/science.1156995 18403674 PMC2396588

[B79] Dumas A. Amiable N. de Rivero Vaccari J. P. Chae J. J. Keane R. W. Lacroix S. (2014). The inflammasome pyrin contributes to pertussis toxin-induced IL-1beta synthesis, neutrophil intravascular crawling and autoimmune encephalomyelitis. *PLoS Pathog.* 10:e1004150. 10.1371/journal.ppat.1004150 24875775 PMC4038594

[B80] Duncan J. A. Gao X. Huang M. T. O’Connor B. P. Thomas C. E. Willingham S. B. (2009). *Neisseria gonorrhoeae* activates the proteinase cathepsin B to mediate the signaling activities of the NLRP3 and ASC-containing inflammasome. *J. Immunol.* 182 6460–6469. 10.4049/jimmunol.0802696 19414800 PMC2722440

[B81] Elinav E. Strowig T. Kau A. L. Henao-Mejia J. Thaiss C. A. Booth C. J. (2011). NLRP6 inflammasome regulates colonic microbial ecology and risk for colitis. *Cell* 145 745–757. 10.1016/j.cell.2011.04.022 21565393 PMC3140910

[B82] Eriksen J. L. Dawson T. M. Dickson D. W. Petrucelli L. (2003). Caught in the act: alpha-synuclein is the culprit in Parkinson’s disease. *Neuron* 40 453–456. 14642269 10.1016/s0896-6273(03)00684-6

[B83] Ermler M. E. Traylor Z. Patel K. Schattgen S. A. Vanaja S. K. Fitzgerald K. A. (2014). Rift Valley fever virus infection induces activation of the NLRP3 inflammasome. *Virology* 449 174–180. 10.1016/j.virol.2013.11.015 24418550 PMC3951897

[B84] Escobar J. Pereda J. Lopez-Rodas G. Sastre J. (2012). Redox signaling and histone acetylation in acute pancreatitis. *Free Radic. Biol. Med.* 52 819–837. 10.1016/j.freeradbiomed.2011.11.009 22178977

[B85] Fabbri M. Garzon R. Cimmino A. Liu Z. Zanesi N. Callegari E. (2007). MicroRNA-29 family reverts aberrant methylation in lung cancer by targeting DNA methyltransferases 3A and 3B. *Proc. Natl. Acad. Sci. U.S.A.* 104 15805–15810. 10.1073/pnas.0707628104 17890317 PMC2000384

[B86] Fang L. Hou Y. An J. Li B. Song M. Wang X. (2016). Genome-wide transcriptional and post-transcriptional regulation of innate immune and defense responses of bovine mammary gland to *Staphylococcus aureus*. *Front. Cell. Infect. Microbiol.* 6:193. 10.3389/fcimb.2016.00193 28083515 PMC5183581

[B87] Farrell P. J. (2018). Epstein-barr virus and cancer. *Annu. Rev. Pathol.* 14 29–53. 10.1146/annurev-pathmechdis-012418-013023 30125149

[B88] Faustin B. Lartigue L. Bruey J. M. Luciano F. Sergienko E. Bailly-Maitre B. (2007). Reconstituted NALP1 inflammasome reveals two-step mechanism of caspase-1 activation. *Mol. Cell* 25 713–724. 10.1016/j.molcel.2007.01.032 17349957

[B89] Fazi F. Rosa A. Fatica A. Gelmetti V. De Marchis M. L. Nervi C. (2005). A minicircuitry comprised of microRNA-223 and transcription factors NFI-A and C/EBPalpha regulates human granulopoiesis. *Cell* 123 819–831. 10.1016/j.cell.2005.09.023 16325577

[B90] Feng X. Luo Q. Wang H. Zhang H. Chen F. (2018). MicroRNA-22 suppresses cell proliferation, migration and invasion in oral squamous cell carcinoma by targeting NLRP3. *J. Cell. Physiol.* 233 6705–6713. 10.1002/jcp.26331 29319163 PMC13482448

[B91] Feng Z. Qi S. Zhang Y. Qi Z. Yan L. Zhou J. (2017). Ly6G+ neutrophil-derived miR-223 inhibits the NLRP3 inflammasome in mitochondrial DAMP-induced acute lung injury. *Cell Death Dis.* 8:e3170. 10.1038/cddis.2017.549 29144508 PMC5775410

[B92] Ferreri A. J. Illerhaus G. Zucca E. Cavalli F. (2010). Flows and flaws in primary central nervous system lymphoma. *Nat. Rev. Clin. Oncol.* 7:472. 20700952 10.1038/nrclinonc.2010.9-c1

[B93] Filipowicz W. Bhattacharyya S. N. Sonenberg N. (2008). Mechanisms of post-transcriptional regulation by microRNAs: are the answers in sight? *Nat. Rev. Genet.* 9 102–114. 10.1038/nrg2290 18197166

[B94] Finger J. N. Lich J. D. Dare L. C. Cook M. N. Brown K. K. Duraiswami C. (2012). Autolytic proteolysis within the function to find domain (FIIND) is required for NLRP1 inflammasome activity. *J. Biol. Chem.* 287 25030–25037. 10.1074/jbc.M112.378323 22665479 PMC3408201

[B95] Fink S. L. Cookson B. T. (2006). Caspase-1-dependent pore formation during pyroptosis leads to osmotic lysis of infected host macrophages. *Cell. Microbiol.* 8 1812–1825. 10.1111/j.1462-5822.2006.00751.x 16824040

[B96] Fiocchi C. (1998). Inflammatory bowel disease: etiology and pathogenesis. *Gastroenterology* 115 182–205. 9649475 10.1016/s0016-5085(98)70381-6

[B97] Franchi L. Amer A. Body-Malapel M. Kanneganti T. D. Ozoren N. Jagirdar R. (2006). Cytosolic flagellin requires Ipaf for activation of caspase-1 and interleukin 1beta in *salmonella*-infected macrophages. *Nat. Immunol.* 7 576–582. 10.1038/ni1346 16648852

[B98] Franchi L. Eigenbrod T. Nunez G. (2009). Cutting edge: TNF-alpha mediates sensitization to ATP and silica via the NLRP3 inflammasome in the absence of microbial stimulation. *J. Immunol.* 183 792–796. 10.4049/jimmunol.0900173 19542372 PMC2754237

[B99] Franchi L. Munoz-Planillo R. Nunez G. (2012). Sensing and reacting to microbes through the inflammasomes. *Nat. Immunol.* 13 325–332. 10.1038/ni.2231 22430785 PMC3449002

[B100] Frantz S. Ducharme A. Sawyer D. Rohde L. E. Kobzik L. Fukazawa R. (2003). Targeted deletion of caspase-1 reduces early mortality and left ventricular dilatation following myocardial infarction. *J. Mol. Cell. Cardiol.* 35 685–694. 12788386 10.1016/s0022-2828(03)00113-5

[B101] Gao W. Yang J. Liu W. Wang Y. Shao F. (2016). Site-specific phosphorylation and microtubule dynamics control Pyrin inflammasome activation. *Proc. Natl. Acad. Sci. U.S.A.* 113 E4857–E4866. 10.1073/pnas.1601700113 27482109 PMC4995971

[B102] Garlanda C. Dinarello C. A. Mantovani A. (2013). The interleukin-1 family: back to the future. *Immunity* 39 1003–1018. 10.1016/j.immuni.2013.11.010 24332029 PMC3933951

[B103] Gasser T. (2009). Molecular pathogenesis of Parkinson disease: insights from genetic studies. *Expert Rev. Mol. Med.* 11:e22. 10.1017/S1462399409001148 19631006

[B104] Giray B. G. Emekdas G. Tezcan S. Ulger M. Serin M. S. Sezgin O. (2014). Profiles of serum microRNAs; miR-125b-5p and miR223-3p serve as novel biomarkers for HBV-positive hepatocellular carcinoma. *Mol. Biol. Rep.* 41 4513–4519. 10.1007/s11033-014-3322-3 24595450

[B105] Glasgow S. M. Laug D. Brawley V. S. Zhang Z. Corder A. Yin Z. (2013). The miR-223/nuclear factor I-A axis regulates glial precursor proliferation and tumorigenesis in the CNS. *J. Neurosci.* 33 13560–13568. 10.1523/JNEUROSCI.0321-13.2013 23946414 PMC3742938

[B106] Gorfu G. Cirelli K. M. Melo M. B. Mayer-Barber K. Crown D. Koller B. H. (2014). Dual role for inflammasome sensors NLRP1 and NLRP3 in murine resistance to *Toxoplasma gondii*. *mBio* 5:e01117-13. 10.1128/mBio.01117-13 24549849 PMC3944820

[B107] Grieco G. E. Cataldo D. Ceccarelli E. Nigi L. Catalano G. Brusco N. (2018). Serum levels of miR-148a and miR-21-5p are increased in type 1 diabetic patients and correlated with markers of bone strength and metabolism. *Noncoding RNA* 4:E37. 10.3390/ncrna4040037 30486455 PMC6315714

[B108] Grishman E. K. White P. C. Savani R. C. (2012). Toll-like receptors, the NLRP3 inflammasome, and interleukin-1beta in the development and progression of type 1 diabetes. *Pediatr. Res.* 71 626–632. 10.1038/pr.2012.24 22337228

[B109] Groslambert M. Py B. F. (2018). Spotlight on the NLRP3 inflammasome pathway. *J. Inflamm. Res.* 11 359–374. 10.2147/JIR.S141220 30288079 PMC6161739

[B110] Guo C. Fu R. Wang S. Huang Y. Li X. Zhou M. (2018). NLRP3 inflammasome activation contributes to the pathogenesis of rheumatoid arthritis. *Clin. Exp. Immunol.* 194 231–243. 10.1111/cei.13167 30277570 PMC6194337

[B111] Guo W. Sun Y. Liu W. Wu X. Guo L. Cai P. (2014). Small molecule-driven mitophagy-mediated NLRP3 inflammasome inhibition is responsible for the prevention of colitis-associated cancer. *Autophagy* 10 972–985. 10.4161/auto.28374 24879148 PMC4091180

[B112] Gurung P. Anand P. K. Malireddi R. K. Vande Walle L. Van Opdenbosch N. Dillon C. P. (2014). FADD and caspase-8 mediate priming and activation of the canonical and noncanonical Nlrp3 inflammasomes. *J. Immunol.* 192 1835–1846. 10.4049/jimmunol.1302839 24453255 PMC3933570

[B113] Haneklaus M. Gerlic M. Kurowska-Stolarska M. Rainey A. A. Pich D. McInnes I. B. (2012). Cutting edge: miR-223 and EBV miR-BART15 regulate the NLRP3 inflammasome and IL-1beta production. *J. Immunol.* 189 3795–3799. 10.4049/jimmunol.1200312 22984081

[B114] Harder J. Franchi L. Munoz-Planillo R. Park J. H. Reimer T. Nunez G. (2009). Activation of the Nlrp3 inflammasome by Streptococcus pyogenes requires streptolysin O and NF-kappa B activation but proceeds independently of TLR signaling and P 2X7 receptor. *J. Immunol.* 183 5823–5829. 10.4049/jimmunol.0900444 19812205 PMC2765568

[B115] He A. Ji R. Shao J. He C. Jin M. Xu Y. (2016). TLR4-MyD88-TRAF6-TAK1 complex-mediated NF-κB activation contribute to the anti-inflammatory effect of V8 in LPS-induced human cervical cancer SiHa cells. *Inflammation* 39 172–181. 10.1007/s10753-015-0236-8 26276130

[B116] He Y. Hara H. Nunez G. (2016). Mechanism and regulation of NLRP3 inflammasome activation. *Trends Biochem. Sci.* 41 1012–1021. 10.1016/j.tibs.2016.09.002 27669650 PMC5123939

[B117] Heil F. Hemmi H. Hochrein H. Ampenberger F. Kirschning C. Akira S. (2004). Species-specific recognition of single-stranded RNA via toll-like receptor 7 and 8. *Science* 303 1526–1529. 14976262 10.1126/science.1093620

[B118] Heilig R. Broz P. (2018). Function and mechanism of the pyrin inflammasome. *Eur. J. Immunol.* 48 230–238. 10.1002/eji.201746947 29148036

[B119] Hilbi H. Moss J. E. Hersh D. Chen Y. Arondel J. Banerjee S. (1998). *Shigella*-induced apoptosis is dependent on caspase-1 which binds to IpaB. *J. Biol. Chem.* 273 32895–32900. 9830039 10.1074/jbc.273.49.32895

[B120] Hise A. G. Tomalka J. Ganesan S. Patel K. Hall B. A. Brown G. D. (2009). An essential role for the NLRP3 inflammasome in host defense against the human fungal pathogen *Candida albicans*. *Cell Host Microbe* 5 487–497. 10.1016/j.chom.2009.05.002 19454352 PMC2824856

[B121] Hoffman H. M. Mueller J. L. Broide D. H. Wanderer A. A. Kolodner R. D. (2001). Mutation of a new gene encoding a putative pyrin-like protein causes familial cold autoinflammatory syndrome and Muckle-Wells syndrome. *Nat. Genet.* 29 301–305. 11687797 10.1038/ng756 PMC4322000

[B122] Hoffman H. M. Throne M. L. Amar N. J. Sebai M. Kivitz A. J. Kavanaugh A. (2008). Efficacy and safety of rilonacept (interleukin-1 Trap) in patients with cryopyrin-associated periodic syndromes: results from two sequential placebo-controlled studies. *Arthritis Rheum.* 58 2443–2452. 10.1002/art.23687 18668535

[B123] Hofmann S. Kubasch A. Ioannidis C. Rösen-Wolff A. Girschick H. Morbach H. (2015). Altered expression of IL-10 family cytokines in monocytes from CRMO patients result in enhanced IL-1β expression and release. *Clin. Immunol.* 161 300–307. 10.1016/j.clim.2015.09.013 26404542

[B124] Hogan D. J. Vincent T. M. Fish S. Marcusson E. G. Bhat B. Chau B. N. (2014). Anti-miRs competitively inhibit microRNAs in Argonaute complexes. *PLoS One* 9:e100951. 10.1371/journal.pone.0100951 24992387 PMC4084633

[B125] Hu Z. Zhou Q. Zhang C. Fan S. Cheng W. Zhao Y. (2015). Structural and biochemical basis for induced self-propagation of NLRC4. *Science* 350 399–404. 10.1126/science.aac5489 26449475

[B126] Huang B. S. Luo Q. Z. Han Y. Huang D. Tang Q. P. Wu L. X. (2017). MiR-223/PAX6 axis regulates glioblastoma stem cell proliferation and the chemo resistance to TMZ via regulating PI3K/Akt pathway. *J. Cell. Biochem.* 118 3452–3461. 10.1002/jcb.26003 28332226

[B127] Huang W. Q. Wei P. Lin R. Q. Huang F. (2017). Protective effects of Microrna-22 against endothelial cell injury by targeting NLRP3 through suppression of the inflammasome signaling pathway in a rat model of coronary heart disease. *Cell. Physiol. Biochem.* 43 1346–1358. 10.1159/000481846 28992621

[B128] Huber S. Gagliani N. Zenewicz L. A. Huber F. J. Bosurgi L. Hu B. (2012). IL-22BP is regulated by the inflammasome and modulates tumorigenesis in the intestine. *Nature* 491 259–263. 10.1038/nature11535 23075849 PMC3493690

[B129] Hutvagner G. Zamore P. D. (2002). A microRNA in a multiple-turnover RNAi enzyme complex. *Science* 297 2056–2060. 10.1126/science.1073827 12154197

[B130] Hwang I. Lee E. Jeon S.-A. Yu J.-W. (2015). Histone deacetylase 6 negatively regulates NLRP3 inflammasome activation. *Biochem. Biophys. Res. Commun.* 467 973–978. 10.1016/j.bbrc.2015.10.033 26471297

[B131] Ichinohe T. Lee H. K. Ogura Y. Flavell R. Iwasaki A. (2009). Inflammasome recognition of influenza virus is essential for adaptive immune responses. *J. Exp. Med.* 206 79–87. 10.1084/jem.20081667 19139171 PMC2626661

[B132] Ichinohe T. Pang I. K. Iwasaki A. (2010). Influenza virus activates inflammasomes via its intracellular M2 ion channel. *Nat. Immunol.* 11 404–410. 10.1038/ni.1861 20383149 PMC2857582

[B133] Inohara N. Nunez G. (2003). NODs: intracellular proteins involved in inflammation and apoptosis. *Nat. Rev. Immunol.* 3 371–382. 12766759 10.1038/nri1086

[B134] Itariu B. K. Stulnig T. M. (2014). Autoimmune aspects of type 2 diabetes mellitus - a mini-review. *Gerontology* 60 189–196. 10.1159/000356747 24457898

[B135] Ito M. Yanagi Y. Ichinohe T. (2012). Encephalomyocarditis virus viroporin 2B activates NLRP3 inflammasome. *PLoS Pathog.* 8:e1002857. 10.1371/journal.ppat.1002857 22916014 PMC3415442

[B136] Jamilloux Y. Magnotti F. Belot A. Henry T. (2018). The pyrin inflammasome: from sensing RhoA GTPases-inhibiting toxins to triggering autoinflammatory syndromes. *Pathog. Dis.* 76:fty020. 10.1093/femspd/fty020 29718184

[B137] Jiang L. Zhang L. Kang K. Fei D. Gong R. Cao Y. (2016). Resveratrol ameliorates LPS-induced acute lung injury via NLRP3 inflammasome modulation. *Biomed. Pharmacother.* 84 130–138. 10.1016/j.biopha.2016.09.020 27643555

[B138] Jin J. Yu Q. Han C. Hu X. Xu S. Wang Q. (2013a). LRRFIP2 negatively regulates NLRP3 inflammasome activation in macrophages by promoting Flightless-I-mediated caspase-1 inhibition. *Nat. Commun.* 4:2075. 10.1038/ncomms3075 23942110 PMC3753543

[B139] Jin T. Curry J. Smith P. Jiang J. Xiao T. S. (2013b). Structure of the NLRP1 caspase recruitment domain suggests potential mechanisms for its association with procaspase-1. *Proteins* 81 1266–1270. 10.1002/prot.24287 23508996 PMC3860829

[B140] Jin T. Perry A. Jiang J. Smith P. Curry J. A. Unterholzner L. (2012). Structures of the HIN domain:DNA complexes reveal ligand binding and activation mechanisms of the AIM2 inflammasome and IFI16 receptor. *Immunity* 36 561–571. 10.1016/j.immuni.2012.02.014 22483801 PMC3334467

[B141] Jin T. Perry A. Smith P. Jiang J. Xiao T. S. (2013c). Structure of the absent in melanoma 2 (AIM2) pyrin domain provides insights into the mechanisms of AIM2 autoinhibition and inflammasome assembly. *J. Biol. Chem.* 288 13225–13235. 10.1074/jbc.M113.468033 23530044 PMC3650362

[B142] Jo E. K. Kim J. K. Shin D. M. Sasakawa C. (2016). Molecular mechanisms regulating NLRP3 inflammasome activation. *Cell. Mol. Immunol.* 13 148–159. 10.1038/cmi.2015.95 26549800 PMC4786634

[B143] Johnnidis J. B. Harris M. H. Wheeler R. T. Stehling-Sun S. Lam M. H. Kirak O. (2008). Regulation of progenitor cell proliferation and granulocyte function by microRNA-223. *Nature* 451 1125–1129. 10.1038/nature06607 18278031

[B144] Joly S. Ma N. Sadler J. J. Soll D. R. Cassel S. L. Sutterwala F. S. (2009). Cutting edge: *Candida albicans* hyphae formation triggers activation of the Nlrp3 inflammasome. *J. Immunol.* 183 3578–3581. 10.4049/jimmunol.0901323 19684085 PMC2739101

[B145] Ju H. Tan J. Y. Cao B. Song M. Q. Tian Z. B. (2018). Effects of miR-223 on colorectal cancer cell proliferation and apoptosis through regulating FoxO3a/BIM. *Eur. Rev. Med. Pharmacol. Sci.* 22 3771–3778. 10.26355/eurrev_201806_15259 29949152

[B146] Kahlenberg J. M. Kaplan M. J. (2014). The inflammasome and lupus: another innate immune mechanism contributing to disease pathogenesis? *Curr. Opin. Rheumatol.* 26 475–481. 10.1097/BOR.0000000000000088 24992143 PMC4153426

[B147] Kahlenberg J. M. Thacker S. G. Berthier C. C. Cohen C. D. Kretzler M. Kaplan M. J. (2011). Inflammasome activation of IL-18 results in endothelial progenitor cell dysfunction in systemic lupus erythematosus. *J. Immunol.* 187 6143–6156. 22058412 10.4049/jimmunol.1101284 PMC3221936

[B148] Kanneganti T. D. Ozoren N. Body-Malapel M. Amer A. Park J. H. Franchi L. (2006). Bacterial RNA and small antiviral compounds activate caspase-1 through cryopyrin/Nalp3. *Nature* 440 233–236. 10.1038/nature04517 16407888

[B149] Karki R. Man S. M. Kanneganti T. D. (2017). Inflammasomes and Cancer. *Cancer Immunol. Res.* 5 94–99. 10.1158/2326-6066.CIR-16-0269 28093447 PMC5593081

[B150] Karunakaran D. Thrush A. B. Nguyen M. A. Richards L. Geoffrion M. Singaravelu R. (2015). Macrophage mitochondrial energy status regulates cholesterol efflux and is enhanced by Anti-miR33 in atherosclerosis. *Circ. Res.* 117 266–278. 10.1161/CIRCRESAHA.117.305624 26002865 PMC4578799

[B151] Kaushik D. K. Gupta M. Kumawat K. L. Basu A. (2012). NLRP3 inflammasome: key mediator of neuroinflammation in murine Japanese encephalitis. *PLoS One* 7:e32270. 10.1371/journal.pone.0032270 22393394 PMC3290554

[B152] Kieff E. Rickinson A. B. (2007). *Epstein–Barr Virus and Its Replication.* Philadelphia, PA: Lippincott Williams, and Wilkins.

[B153] Kim D. H. Kim S. M. Lee B. Lee E. K. Chung K. W. Moon K. M. (2017). Effect of betaine on hepatic insulin resistance through FOXO1-induced NLRP3 inflammasome. *J. Nutr. Biochem.* 45 104–114. 10.1016/j.jnutbio.2017.04.014 28499186

[B154] Kitamura A. Sasaki Y. Abe T. Kano H. Yasutomo K. (2014). An inherited mutation in NLRC4 causes autoinflammation in human and mice. *J. Exp. Med.* 211 2385–2396. 10.1084/jem.20141091 25385754 PMC4235634

[B155] Kleemann R. Zadelaar S. Kooistra T. (2008). Cytokines and atherosclerosis: a comprehensive review of studies in mice. *Cardiovasc. Res.* 79 360–376. 10.1093/cvr/cvn120 18487233 PMC2492729

[B156] Kloppel G. Lohr M. Habich K. Oberholzer M. Heitz P. U. (1985). Islet pathology and the pathogenesis of type 1 and type 2 diabetes mellitus revisited. *Surv. Synth. Pathol. Res.* 4 110–125. 3901180 10.1159/000156969

[B157] Kofahi H. Taylor N. Hirasawa K. Grant M. Russell R. (2016). Hepatitis C virus infection of cultured human hepatoma cells causes apoptosis and pyroptosis in both infected and bystander cells. *Sci. Rep.* 6:37433. 10.1038/srep37433 27974850 PMC5156923

[B158] Koizumi Y. Toma C. Higa N. Nohara T. Nakasone N. Suzuki T. (2012). Inflammasome activation via intracellular NLRs triggered by bacterial infection. *Cell. Microbiol.* 14 149–154. 10.1111/j.1462-5822.2011.01707.x 21995284

[B159] Kolb R. Liu G. H. Janowski A. M. Sutterwala F. S. Zhang W. (2014). Inflammasomes in cancer: a double-edged sword. *Protein Cell* 5 12–20. 10.1007/s13238-013-0001-4 24474192 PMC3938856

[B160] Kortmann J. Brubaker S. W. Monack D. M. (2015). Cutting edge: inflammasome activation in primary human macrophages is dependent on flagellin. *J. Immunol.* 195 815–819. 10.4049/jimmunol.1403100 26109648 PMC4505955

[B161] Krek A. Grun D. Poy M. N. Wolf R. Rosenberg L. Epstein E. J. (2005). Combinatorial microRNA target predictions. *Nat. Genet.* 37 495–500. 10.1038/ng1536 15806104

[B162] Kriek J.-M. Jaumdally S. Z. Masson L. Little F. Mbulawa Z. Gumbi P. P. (2016). Female genital tract inflammation, HIV co-infection and persistent mucosal Human Papillomavirus (HPV) infections. *Virology* 493 247–254. 10.1016/j.virol.2016.03.022 27065342

[B163] Lachmann H. J. Kone-Paut I. Kuemmerle-Deschner J. B. Leslie K. S. Hachulla E. Quartier P. (2009). Use of canakinumab in the cryopyrin-associated periodic syndrome. *N. Engl. J. Med.* 360 2416–2425. 10.1056/NEJMoa0810787 19494217

[B164] Lara-Tejero M. Sutterwala F. S. Ogura Y. Grant E. P. Bertin J. Coyle A. J. (2006). Role of the caspase-1 inflammasome in *Salmonella* typhimurium pathogenesis. *J. Exp. Med.* 203 1407–1412. 10.1084/jem.20060206 16717117 PMC2118315

[B165] Larsen C. M. Faulenbach M. Vaag A. Volund A. Ehses J. A. Seifert B. (2007). Interleukin-1-receptor antagonist in type 2 diabetes mellitus. *N. Engl. J. Med.* 356 1517–1526. 10.1056/NEJMoa065213 17429083

[B166] Laudato S. Patil N. Abba M. L. Leupold J. H. Benner A. Gaiser T. (2017). P53-induced miR-30e-5p inhibits colorectal cancer invasion and metastasis by targeting ITGA6 and ITGB1. *Int. J. Cancer* 141 1879–1890. 10.1002/ijc.30854 28656629

[B167] Leipe J. Grunke M. Dechant C. Reindl C. Kerzendorf U. Schulze-Koops H. (2010). Role of Th17 cells in human autoimmune arthritis. *Arthritis Rheum.* 62 2876–2885. 10.1002/art.27622 20583102

[B168] Levy M. Thaiss C. A. Zeevi D. Dohnalova L. Zilberman-Schapira G. Mahdi J. A. (2015). Microbiota-modulated metabolites shape the intestinal microenvironment by regulating NLRP6 inflammasome signaling. *Cell* 163 1428–1443. 10.1016/j.cell.2015.10.048 26638072 PMC5665753

[B169] Lewis B. P. Burge C. B. Bartel D. P. (2005). Conserved seed pairing, often flanked by adenosines, indicates that thousands of human genes are microRNA targets. *Cell* 120 15–20. 10.1016/j.cell.2004.12.035 15652477

[B170] Li B. Song Y. Liu T.-J. Cui Y.-B. Jiang Y. Xie Z.-S. (2013). miRNA-22 suppresses colon cancer cell migration and invasion by inhibiting the expression of T-cell lymphoma invasion and metastasis 1 and matrix metalloproteinases 2 and 9. *Oncol. Rep.* 29 1932–1938. 10.3892/or.2013.2300 23440286

[B171] Li D. Yang H. Ma J. Luo S. Chen S. Gu Q. (2018). MicroRNA-30e regulates neuroinflammation in MPTP model of Parkinson’s disease by targeting Nlrp3. *Hum. Cell* 31 106–115. 10.1007/s13577-017-0187-5 29274035 PMC5852205

[B172] Li P. Zhong X. Li J. Liu H. Ma X. He R. (2018). MicroRNA-30c-5p inhibits NLRP3 inflammasome-mediated endothelial cell pyroptosis through FOXO3 down-regulation in atherosclerosis. *Biochem. Biophys. Res. Commun.* 503 2833–2840. 10.1016/j.bbrc.2018.08.049 30119891

[B173] Li S. Liang X. Ma L. Shen L. Li T. Zheng L. (2018). MiR-22 sustains NLRP3 expression and attenuates H. *pylori*-induced gastric carcinogenesis. *Oncogene* 37 884–896. 10.1038/onc.2017.381 29059152

[B174] Li W. Sun W. Liu L. Yang F. Li Y. Chen Y. (2010). IL-32: a host proinflammatory factor against influenza viral replication is upregulated by aberrant epigenetic modifications during influenza A virus infection. *J. Immunol.* 185 5056–5065. 10.4049/jimmunol.0902667 20889550

[B175] Li W. B. Chen H. Y. Zhang W. Yan W. Shi R. Li S. W. (2013). Relationship between magnetic resonance imaging features and miRNA gene expression in patients with glioblastoma multiforme. *Chin. Med. J.* 126 2881–2885. 23924460

[B176] Li X. Zhang Y. Zhang H. Liu X. Gong T. Li M. (2011). miRNA-223 promotes gastric cancer invasion and metastasis by targeting tumor suppressor EPB41L3. *Mol. Cancer Res.* 9 824–833. 10.1158/1541-7786.MCR-10-0529 21628394

[B177] Li Z. Rana T. M. (2014). Therapeutic targeting of microRNAs: current status and future challenges. *Nat. Rev. Drug Discov.* 13 622–638. 10.1038/nrd4359 25011539

[B178] Li Z. Yang C. S. Nakashima K. Rana T. M. (2011). Small RNA-mediated regulation of iPS cell generation. *EMBO J.* 30 823–834. 10.1038/emboj.2011.2 21285944 PMC3049216

[B179] Lich J. D. Williams K. L. Moore C. B. Arthur J. C. Davis B. K. Taxman D. J. (2007). Monarch-1 suppresses non-canonical NF-kappaB activation and p52-dependent chemokine expression in monocytes. *J. Immunol.* 178 1256–1260. 17237370 10.4049/jimmunol.178.3.1256

[B180] Lindow M. Kauppinen S. (2012). Discovering the first microRNA-targeted drug. *J. Cell Biol.* 199 407–412. 10.1083/jcb.201208082 23109665 PMC3483128

[B181] Lissner D. Siegmund B. (2011). The multifaceted role of the inflammasome in inflammatory bowel diseases. *Sci. World J.* 11 1536–1547. 10.1100/tsw.2011.139 21805022 PMC5596529

[B182] Liu C. C. Huang Z. X. Li X. Shen K. F. Liu M. Ouyang H. D. (2018). Upregulation of NLRP3 via STAT3-dependent histone acetylation contributes to painful neuropathy induced by bortezomib. *Exp. Neurol.* 302 104–111. 10.1016/j.expneurol.2018.01.011 29339053

[B183] Liu D. Zeng X. Li X. Mehta J. L. Wang X. (2018). Role of NLRP3 inflammasome in the pathogenesis of cardiovascular diseases. *Basic Res. Cardiol.* 113:5. 10.1007/s00395-017-0663-9 29224086

[B184] Liu W. Yin Y. Zhou Z. He M. Dai Y. (2014). OxLDL-induced IL-1beta secretion promoting foam cells formation was mainly via CD36 mediated ROS production leading to NLRP3 inflammasome activation. *Inflamm. Res.* 63 33–43. 24121974 10.1007/s00011-013-0667-3

[B185] Liu Y. Chen X. Cheng R. Yang F. Yu M. Wang C. (2018). The Jun/miR-22/HuR regulatory axis contributes to tumourigenesis in colorectal cancer. *Mol. Cancer* 17:11. 10.1186/s12943-017-0751-3 29351796 PMC5775639

[B186] Lu A. Magupalli V. G. Ruan J. Yin Q. Atianand M. K. Vos M. R. (2014). Unified polymerization mechanism for the assembly of ASC-dependent inflammasomes. *Cell* 156 1193–1206. 10.1016/j.cell.2014.02.008 24630722 PMC4000066

[B187] Lu M. Sun X.-L. Qiao C. Liu Y. Ding J.-H. Hu G. (2014). Uncoupling protein 2 deficiency aggravates astrocytic endoplasmic reticulum stress and nod-like receptor protein 3 inflammasome activation. *Neurobiol. Aging* 35 421–430. 10.1016/j.neurobiolaging.2013.08.015 24041971

[B188] Lupfer C. Malik A. Kanneganti T. D. (2015). Inflammasome control of viral infection. *Curr. Opin. Virol.* 12 38–46. 10.1016/j.coviro.2015.02.007 25771504 PMC4470791

[B189] Lust J. A. Lacy M. Q. Zeldenrust S. R. Dispenzieri A. Gertz M. A. Witzig T. E. (2009). Induction of a chronic disease state in patients with smoldering or indolent multiple myeloma by targeting interleukin 1{beta}-induced interleukin 6 production and the myeloma proliferative component. *Mayo Clin. Proc.* 84 114–122. 10.4065/84.2.114 19181644 PMC2664581

[B190] Ma C.-H. Kang L.-L. Ren H.-M. Zhang D.-M. Kong L.-D. (2015). Simiao pill ameliorates renal glomerular injury via increasing Sirt1 expression and suppressing NF-κB/NLRP3 inflammasome activation in high fructose-fed rats. *J. Ethnopharmacol.* 172 108–117. 10.1016/j.jep.2015.06.015 26117533

[B191] Mamik M. K. Power C. (2017). Inflammasomes in neurological diseases: emerging pathogenic and therapeutic concepts. *Brain* 140 2273–2285. 10.1093/brain/awx133 29050380

[B192] Manikandan M. Deva Magendhra Rao A. K. Arunkumar G. Manickavasagam M. Rajkumar K. S. Rajaraman R. (2016). Oral squamous cell carcinoma: microRNA expression profiling and integrative analyses for elucidation of tumourigenesis mechanism. *Mol. Cancer* 15:28. 10.1186/s12943-016-0512-8 27056547 PMC4823852

[B193] Mao L. Zhang L. Li H. Chen W. Wang H. Wu S. (2014). Pathogenic fungus Microsporum canis activates the NLRP3 inflammasome. *Infect. Immun.* 82 882–892. 10.1128/IAI.01097-13 24478101 PMC3911390

[B194] Mariathasan S. Newton K. Monack D. M. Vucic D. French D. M. Lee W. P. (2004). Differential activation of the inflammasome by caspase-1 adaptors ASC and Ipaf. *Nature* 430 213–218. 10.1038/nature02664 15190255

[B195] Mariathasan S. Weiss D. S. Newton K. McBride J. O’Rourke K. Roose-Girma M. (2006). Cryopyrin activates the inflammasome in response to toxins and ATP. *Nature* 440 228–232. 10.1038/nature04515 16407890

[B196] Martinon F. Burns K. Tschopp J. (2002). The inflammasome: a molecular platform triggering activation of inflammatory caspases and processing of proIL-beta. *Mol. Cell* 10 417–426. 12191486 10.1016/s1097-2765(02)00599-3

[B197] Martinon F. Petrilli V. Mayor A. Tardivel A. Tschopp J. (2006). Gout-associated uric acid crystals activate the NALP3 inflammasome. *Nature* 440 237–241. 10.1038/nature04516 16407889

[B198] Master S. S. Rampini S. K. Davis A. S. Keller C. Ehlers S. Springer B. (2008). *Mycobacterium tuberculosis* prevents inflammasome activation. *Cell Host Microbe* 3 224–232. 10.1016/j.chom.2008.03.003 18407066 PMC3657562

[B199] Masters S. L. Simon A. Aksentijevich I. Kastner D. L. (2009). Horror autoinflammaticus: the molecular pathophysiology of autoinflammatory disease (^∗^). *Annu. Rev. Immunol.* 27 621–668. 10.1146/annurev.immunol.25.022106.141627 19302049 PMC2996236

[B200] McAfoose J. Baune B. T. (2009). Evidence for a cytokine model of cognitive function. *Neurosci. Biobehav. Rev.* 33 355–366. 10.1016/j.neubiorev.2008.10.005 18996146

[B201] McElvania Tekippe E. Allen I. C. Hulseberg P. D. Sullivan J. T. McCann J. R. Sandor M. (2010). Granuloma formation and host defense in chronic *Mycobacterium tuberculosis* infection requires PYCARD/ASC but not NLRP3 or caspase-1. *PLoS One* 5:e12320. 10.1371/journal.pone.0012320 20808838 PMC2924896

[B202] McInnes I. B. Schett G. (2011). The pathogenesis of rheumatoid arthritis. *N. Engl. J. Med.* 365 2205–2219. 10.1056/NEJMra1004965 22150039

[B203] McKenzie B. A. Mamik M. K. Saito L. B. Boghozian R. Monaco M. C. Major E. O. (2018). Caspase-1 inhibition prevents glial inflammasome activation and pyroptosis in models of multiple sclerosis. *Proc. Natl. Acad. Sci. U.S.A.* 115 E6065–E6074. 10.1073/pnas.1722041115 29895691 PMC6042136

[B204] Melehani J. H. Duncan J. A. (2016). Inflammasome activation can mediate tissue-specific pathogenesis or protection in *Staphylococcus aureus* infection. *Curr. Top. Microbiol. Immunol.* 397 257–282. 10.1007/978-3-319-41171-2_13 27460814 PMC5145311

[B205] Mende R. Vincent F. B. Kandane-Rathnayake R. Koelmeyer R. Lin E. Chang J. (2018). Analysis of serum interleukin (IL)-1beta and IL-18 in systemic lupus erythematosus. *Front. Immunol.* 9:1250. 10.3389/fimmu.2018.01250 29930551 PMC5999794

[B206] Merhautova J. Demlova R. Slaby O. (2016). MicroRNA-based therapy in animal models of selected gastrointestinal cancers. *Front. Pharmacol.* 7:329. 10.3389/fphar.2016.00329 27729862 PMC5037200

[B207] Miao E. A. Alpuche-Aranda C. M. Dors M. Clark A. E. Bader M. W. Miller S. I. (2006). Cytoplasmic flagellin activates caspase-1 and secretion of interleukin 1beta via Ipaf. *Nat. Immunol.* 7 569–575. 10.1038/ni1344 16648853

[B208] Miao E. A. Leaf I. A. Treuting P. M. Mao D. P. Dors M. Sarkar A. (2010). Caspase-1-induced pyroptosis is an innate immune effector mechanism against intracellular bacteria. *Nat. Immunol.* 11 1136–1142. 10.1038/ni.1960 21057511 PMC3058225

[B209] Miao H. Ou J. Ma Y. Guo F. Yang Z. Wiggins M. (2014). Macrophage CGI-58 deficiency activates ROS-inflammasome pathway to promote insulin resistance in mice. *Cell Rep.* 7 223–235. 10.1016/j.celrep.2014.02.047 24703845 PMC4040312

[B210] Minkiewicz J. de Rivero Vaccari J. P. Keane R. W. (2013). Human astrocytes express a novel NLRP2 inflammasome. *Glia* 61 1113–1121. 10.1002/glia.22499 23625868

[B211] Moles R. (2017). MicroRNAs-based therapy: a novel and promising strategy for cancer treatment. *Microrna* 6 102–109. 10.2174/2211536606666170710183039 28699479

[B212] Mourelatos Z. Dostie J. Paushkin S. Sharma A. Charroux B. Abel L. (2002). miRNPs: a novel class of ribonucleoproteins containing numerous microRNAs. *Genes Dev.* 16 720–728. 10.1101/gad.974702 11914277 PMC155365

[B213] Munoz-Planillo R. Franchi L. Miller L. S. Nunez G. (2009). A critical role for hemolysins and bacterial lipoproteins in *Staphylococcus aureus*-induced activation of the Nlrp3 inflammasome. *J. Immunol.* 183 3942–3948. 10.4049/jimmunol.0900729 19717510 PMC2762867

[B214] Muruve D. A. Petrilli V. Zaiss A. K. White L. R. Clark S. A. Ross P. J. (2008). The inflammasome recognizes cytosolic microbial and host DNA and triggers an innate immune response. *Nature* 452 103–107. 10.1038/nature06664 18288107

[B215] Muxel S. M. Acuna S. M. Aoki J. I. Zampieri R. A. Floeter-Winter L. M. (2018). Toll-like receptor and miRNA-let-7e expression alter the inflammatory response in leishmania amazonensis-infected macrophages. *Front. Immunol.* 9:2792. 10.3389/fimmu.2018.02792 30555476 PMC6283264

[B216] Nahid M. A. Satoh M. Chan E. K. (2011). MicroRNA in TLR signaling and endotoxin tolerance. *Cell. Mol. Immunol.* 8 388–403. 10.1038/cmi.2011.26 21822296 PMC3618661

[B217] Nakamura A. Osonoi T. Terauchi Y. (2010). Relationship between urinary sodium excretion and pioglitazone-induced edema. *J. Diabetes Investig.* 1 208–211. 10.1111/j.2040-1124.2010.00046.x 24843434 PMC4020723

[B218] Nathan C. F. Murray H. W. Wiebe M. E. Rubin B. Y. (1983). Identification of interferon-gamma as the lymphokine that activates human macrophage oxidative metabolism and antimicrobial activity. *J. Exp. Med.* 158 670–689. 6411853 10.1084/jem.158.3.670 PMC2187114

[B219] Negash A. A. Ramos H. J. Crochet N. Lau D. T. Doehle B. Papic N. (2013). IL-1beta production through the NLRP3 inflammasome by hepatic macrophages links hepatitis C virus infection with liver inflammation and disease. *PLoS Pathog.* 9:e1003330. 10.1371/journal.ppat.1003330 23633957 PMC3635973

[B220] Neudecker V. Haneklaus M. Jensen O. Khailova L. Masterson J. C. Tye H. (2017). Myeloid-derived miR-223 regulates intestinal inflammation via repression of the NLRP3 inflammasome. *J. Exp. Med.* 214 1737–1752. 10.1084/jem.20160462 28487310 PMC5460990

[B221] Nordlander S. Pott J. Maloy K. J. (2014). NLRC4 expression in intestinal epithelial cells mediates protection against an enteric pathogen. *Mucosal Immunol.* 7 775–785. 10.1038/mi.2013.95 24280936 PMC4020766

[B222] Okamoto M. Liu W. Luo Y. Tanaka A. Cai X. Norris D. A. (2010). Constitutively active inflammasome in human melanoma cells mediating autoinflammation via caspase-1 processing and secretion of interleukin-1beta. *J. Biol. Chem.* 285 6477–6488. 10.1074/jbc.M109.064907 20038581 PMC2825443

[B223] Omenetti A. Carta S. Delfino L. Martini A. Gattorno M. Rubartelli A. (2014). Increased NLRP3-dependent interleukin 1beta secretion in patients with familial Mediterranean fever: correlation with MEFV genotype. *Ann. Rheum. Dis.* 73 462–469. 10.1136/annrheumdis-2012-202774 23505242

[B224] Ona V. O. Li M. Vonsattel J. P. Andrews L. J. Khan S. Q. Chung W. M. (1999). Inhibition of caspase-1 slows disease progression in a mouse model of Huntington’s disease. *Nature* 399 263–267. 10.1038/20446 10353249

[B225] Østerud B. Bjørklid E. (2003). Role of monocytes in atherogenesis. *Physiol. Rev.* 83 1069–1112. 14506301 10.1152/physrev.00005.2003

[B226] Ouimet M. Ediriweera H. N. Gundra U. M. Sheedy F. J. Ramkhelawon B. Hutchison S. B. (2015). MicroRNA-33-dependent regulation of macrophage metabolism directs immune cell polarization in atherosclerosis. *J. Clin. Invest.* 125 4334–4348. 10.1172/JCI81676 26517695 PMC4665799

[B227] Oyanguren-Desez O. Rodriguez-Antiguedad A. Villoslada P. Domercq M. Alberdi E. Matute C. (2011). Gain-of-function of P2X7 receptor gene variants in multiple sclerosis. *Cell Calcium* 50 468–472. 10.1016/j.ceca.2011.08.002 21906809

[B228] Perz J. F. Armstrong G. L. Farrington L. A. Hutin Y. J. Bell B. P. (2006). The contributions of hepatitis B virus and hepatitis C virus infections to cirrhosis and primary liver cancer worldwide. *J. Hepatol.* 45 529–538. 10.1016/j.jhep.2006.05.013 16879891

[B229] Peschansky V. J. Wahlestedt C. (2014). Non-coding RNAs as direct and indirect modulators of epigenetic regulation. *Epigenetics* 9 3–12. 10.4161/epi.27473 24739571 PMC3928183

[B230] Piccini A. Carta S. Tassi S. Lasiglie D. Fossati G. Rubartelli A. (2008). ATP is released by monocytes stimulated with pathogen-sensing receptor ligands and induces IL-1beta and IL-18 secretion in an autocrine way. *Proc. Natl. Acad. Sci. U.S.A.* 105 8067–8072. 10.1073/pnas.0709684105 18523012 PMC2430360

[B231] Polytarchou C. Oikonomopoulos A. Mahurkar S. Touroutoglou A. Koukos G. Hommes D. W. (2015). Assessment of circulating MicroRNAs for the diagnosis and disease activity evaluation in patients with ulcerative colitis by using the nanostring technology. *Inflamm. Bowel Dis.* 21 2533–2539. 10.1097/MIB.0000000000000547 26313695

[B232] Rauch I. Deets K. A. Ji D. X. von Moltke J. Tenthorey J. L. Lee A. Y. (2017). NAIP-NLRC4 Inflammasomes Coordinate Intestinal Epithelial Cell Expulsion with Eicosanoid and IL-18 Release via Activation of Caspase-1 and -8. *Immunity* 46 649–659. 10.1016/j.immuni.2017.03.016 28410991 PMC5476318

[B233] Rayamajhi M. Zak D. E. Chavarria-Smith J. Vance R. E. Miao E. A. (2013). Cutting edge: mouse NAIP1 detects the type III secretion system needle protein. *J. Immunol.* 191 3986–3989. 10.4049/jimmunol.1301549 24043898 PMC3819181

[B234] Rickinson A. B. Kieff E. (2007). “Epstein–Barr virus,” in *Fields Virology*, 5th Edn, eds Knipe D. M. Howley P. M. (Philadelphia, PA: Lippincott Williams, Wilkins).

[B235] Rose T. Dorner T. (2017). Drivers of the immunopathogenesis in systemic lupus erythematosus. *Best Pract. Res. Clin. Rheumatol.* 31 321–333. 10.1016/j.berh.2017.09.007 29224674

[B236] Ross R. (1993). The pathogenesis of atherosclerosis: a perspective for the 1990s. *Nature* 362 801. 10.1038/362801a0 8479518

[B237] Ruvkun G. (2001). Molecular biology. Glimpses of a tiny RNA world. *Science* 294 797–799. 10.1126/science.1066315 11679654

[B238] Ryan J. L. Jones R. J. Kenney S. C. Rivenbark A. G. Tang W. Knight E. R. (2010). Epstein-Barr virus-specific methylation of human genes in gastric cancer cells. *Infect. Agent Cancer* 5:27. 10.1186/1750-9378-5-27 21194482 PMC3023757

[B239] Sagulenko V. Thygesen S. J. Sester D. P. Idris A. Cridland J. A. Vajjhala P. R. (2013). AIM2 and NLRP3 inflammasomes activate both apoptotic and pyroptotic death pathways via ASC. *Cell Death Differ.* 20 1149–1160. 10.1038/cdd.2013.37 23645208 PMC3741496

[B240] Said-Sadier N. Padilla E. Langsley G. Ojcius D. M. (2010). Aspergillus fumigatus stimulates the NLRP3 inflammasome through a pathway requiring ROS production and the Syk tyrosine kinase. *PLoS One* 5:e10008. 10.1371/journal.pone.0010008 20368800 PMC2848854

[B241] Sandstrom A. Mitchell P. S. Goers L. Mu E. W. Lesser C. F. Vance R. E. (2019). Functional degradation: a mechanism of NLRP1 inflammasome activation by diverse pathogen enzymes. *Science* 364:eaau1330. 10.1126/science.aau1330 30872533 PMC6532986

[B242] Sansonetti P. J. Phalipon A. Arondel J. Thirumalai K. Banerjee S. Akira S. (2000). Caspase-1 activation of IL-1beta and IL-18 are essential for *Shigella* flexneri-induced inflammation. *Immunity* 12 581–590. 10843390 10.1016/s1074-7613(00)80209-5

[B243] Savage C. D. Lopez-Castejon G. Denes A. Brough D. (2012). NLRP3-inflammasome activating DAMPs stimulate an inflammatory response in glia in the absence of priming which contributes to brain inflammation after injury. *Front. Immunol.* 3:288. 23024646 10.3389/fimmu.2012.00288 PMC3444764

[B244] Schaner P. Richards N. Wadhwa A. Aksentijevich I. Kastner D. Tucker P. (2001). Episodic evolution of pyrin in primates: human mutations recapitulate ancestral amino acid states. *Nat. Genet.* 27 318–321. 10.1038/85893 11242116

[B245] Schnitger A. K. Machova A. Mueller R. U. Androulidaki A. Schermer B. Pasparakis M. (2011). Listeria monocytogenes infection in macrophages induces vacuolar-dependent host miRNA response. *PLoS One* 6:e27435. 10.1371/journal.pone.0027435 22114673 PMC3219661

[B246] Schroder K. Tschopp J. (2010). The inflammasomes. *Cell* 140 821–832. 10.1016/j.cell.2010.01.040 20303873

[B247] Schroder K. Zhou R. Tschopp J. (2010). The NLRP3 inflammasome: a sensor for metabolic danger? *Science* 327 296–300. 10.1126/science.1184003 20075245

[B248] Scott D. L. Wolfe F. Huizinga T. W. (2010). Rheumatoid arthritis. *Lancet* 376 1094–1108. 10.1016/S0140-6736(10)60826-4 20870100

[B249] Sellin M. E. Muller A. A. Felmy B. Dolowschiak T. Diard M. Tardivel A. (2014). Epithelium-intrinsic NAIP/NLRC4 inflammasome drives infected enterocyte expulsion to restrict *Salmonella* replication in the intestinal mucosa. *Cell Host Microbe* 16 237–248. 10.1016/j.chom.2014.07.001 25121751

[B250] Seo S. U. Kamada N. Munoz-Planillo R. Kim Y. G. Kim D. Koizumi Y. (2015). Distinct commensals induce interleukin-1beta via NLRP3 inflammasome in inflammatory monocytes to promote intestinal inflammation in response to injury. *Immunity* 42 744–755. 10.1016/j.immuni.2015.03.004 25862092 PMC4408263

[B251] Shahrara S. Pickens S. R. Dorfleutner A. Pope R. M. (2009). IL-17 induces monocyte migration in rheumatoid arthritis. *J. Immunol.* 182 3884–3891. 10.4049/jimmunol.0802246 19265168 PMC2811490

[B252] Shashkin P. Dragulev B. Ley K. (2005). Macrophage differentiation to foam cells. *Curr. Pharm. Des.* 11 3061–3072. 16178764 10.2174/1381612054865064

[B253] Sheedy F. J. Palsson-McDermott E. Hennessy E. J. Martin C. O’Leary J. J. Ruan Q. (2010). Negative regulation of TLR4 via targeting of the proinflammatory tumor suppressor PDCD4 by the microRNA miR-21. *Nat. Immunol.* 11 141–147. 10.1038/ni.1828 19946272

[B254] Shouval D. S. Biswas A. Kang Y. H. Griffith A. E. Konnikova L. Mascanfroni I. D. (2016). Interleukin 1beta mediates intestinal inflammation in mice and patients with interleukin 10 receptor deficiency. *Gastroenterology* 151 1100–1104. 10.1053/j.gastro.2016.08.055 27693323 PMC5124405

[B255] Siegmund B. Lehr H. A. Fantuzzi G. Dinarello C. A. (2001). IL-1 beta -converting enzyme (caspase-1) in intestinal inflammation. *Proc. Natl. Acad. Sci. U.S.A.* 98 13249–13254. 11606779 10.1073/pnas.231473998 PMC60856

[B256] Song L. Pei L. Yao S. Wu Y. Shang Y. (2017). NLRP3 inflammasome in neurological diseases, from functions to therapies. *Front. Cell. Neurosci.* 11:63 10.3389/fncel.2017.00063 PMC5343070 28337127

[B257] Stanley S. A. Raghavan S. Hwang W. W. Cox J. S. (2003). Acute infection and macrophage subversion by *Mycobacterium tuberculosis* require a specialized secretion system. *Proc. Natl. Acad. Sci. U.S.A.* 100 13001–13006. 10.1073/pnas.2235593100 14557536 PMC240734

[B258] Stein M. Keshav S. Harris N. Gordon S. (1992). Interleukin 4 potently enhances murine macrophage mannose receptor activity: a marker of alternative immunologic macrophage activation. *J. Exp. Med.* 176 287–292. 1613462 10.1084/jem.176.1.287 PMC2119288

[B259] Stutz A. Horvath G. L. Monks B. G. Latz E. (2013). ASC speck formation as a readout for inflammasome activation. *Methods Mol. Biol.* 1040 91–101. 10.1007/978-1-62703-523-1_8 23852599

[B260] Suh H.-S. Zhao M.-L. Derico L. Choi N. Lee S. C. (2013). Insulin-like growth factor 1 and 2 (IGF1, IGF2) expression in human microglia: differential regulation by inflammatory mediators. *J. Neuroinflammation* 10:805. 10.1186/1742-2094-10-37 23497056 PMC3607995

[B261] Tabas I. (2005). Consequences and therapeutic implications of macrophage apoptosis in atherosclerosis: the importance of lesion stage and phagocytic efficiency. *Arterioscler. Thromb. Vasc. Biol.* 25 2255–2264. 16141399 10.1161/01.ATV.0000184783.04864.9f

[B262] Tagawa T. Albanese M. Bouvet M. Moosmann A. Mautner J. Heissmeyer V. (2016). Epstein-Barr viral miRNAs inhibit antiviral CD4+ T cell responses targeting IL-12 and peptide processing. *J. Exp. Med.* 213 2065–2080. 10.1084/jem.20160248 27621419 PMC5030804

[B263] Tan Y. Yu L. Zhang C. Chen K. Lu J. Tan L. (2018). miRNA-146a attenuates inflammation in an in vitro spinal cord injury model via inhibition of TLR4 signaling. *Exp. Ther. Med.* 16 3703–3709. 10.3892/etm.2018.6645 30233729 PMC6143872

[B264] Tarassishin L. Lim J. Weatherly D. B. Angeletti R. H. Lee S. C. (2014). Interleukin-1-induced changes in the glioblastoma secretome suggest its role in tumor progression. *J. Proteomics* 99 152–168. 10.1016/j.jprot.2014.01.024 24503185 PMC3977979

[B265] Tenthorey J. L. Kofoed E. M. Daugherty M. D. Malik H. S. Vance R. E. (2014). Molecular basis for specific recognition of bacterial ligands by NAIP/NLRC4 inflammasomes. *Mol. Cell* 54 17–29. 10.1016/j.molcel.2014.02.018 24657167 PMC3988258

[B266] Thomas P. G. Dash P. Aldridge JR Jr Ellebedy A. H. Reynolds C. Funk A. J. (2009). The intracellular sensor NLRP3 mediates key innate and healing responses to influenza A virus via the regulation of caspase-1. *Immunity* 30 566–575. 10.1016/j.immuni.2009.02.006 19362023 PMC2765464

[B267] Thompson M. G. Larson M. Vidrine A. Barrios K. Navarro F. Meyers K. (2015). FOXO3–NF-κB RelA protein complexes reduce proinflammatory cell signaling and function. *J. Immunol.* 195 5637–5647. 10.4049/jimmunol.1501758 26561547 PMC4670825

[B268] Ting J. P. Lovering R. C. Alnemri E. S. Bertin J. Boss J. M. Davis B. K. (2008). The NLR gene family: a standard nomenclature. *Immunity* 28 285–287. 10.1016/j.immuni.2008.02.005 18341998 PMC2630772

[B269] Triantafilou K. Kar S. Vakakis E. Kotecha S. Triantafilou M. (2013a). Human respiratory syncytial virus viroporin SH: a viral recognition pathway used by the host to signal inflammasome activation. *Thorax* 68 66–75. 10.1136/thoraxjnl-2012-202182 23229815

[B270] Triantafilou K. Kar S. van Kuppeveld F. J. Triantafilou M. (2013b). Rhinovirus-induced calcium flux triggers NLRP3 and NLRC5 activation in bronchial cells. *Am. J. Respir. Cell Mol. Biol.* 49 923–934. 10.1165/rcmb.2013-0032OC 23815151

[B271] Tsokos G. C. (2011). Systemic lupus erythematosus. *N. Engl. J. Med.* 365 2110–2121. 10.1056/NEJMra1100359 22129255

[B272] Tsuji N. M. Tsutsui H. Seki E. Kuida K. Okamura H. Nakanishi K. (2004). Roles of caspase-1 in Listeria infection in mice. *Int. Immunol.* 16 335–343. 14734619 10.1093/intimm/dxh041

[B273] Tuddenham L. Wheeler G. Ntounia-Fousara S. Waters J. Hajihosseini M. K. Clark I. (2006). The cartilage specific microRNA-140 targets histone deacetylase 4 in mouse cells. *FEBS Lett.* 580 4214–4217. 10.1016/j.febslet.2006.06.080 16828749

[B274] Valderrama J. A. Riestra A. M. Gao N. J. LaRock C. N. Gupta N. Ali S. R. (2017). Group A streptococcal M protein activates the NLRP3 inflammasome. *Nat. Microbiol.* 2 1425–1434. 10.1038/s41564-017-0005-6 28784982 PMC5750061

[B275] van der Ree M. H. van der Meer A. J. van Nuenen A. C. de Bruijne J. Ottosen S. Janssen H. L. (2016). Miravirsen dosing in chronic hepatitis C patients results in decreased microRNA-122 levels without affecting other microRNAs in plasma. *Aliment. Pharmacol. Ther.* 43 102–113. 10.1111/apt.13432 26503793

[B276] Vento-Tormo R. Álvarez-Errico D. Garcia-Gomez A. Hernández-Rodríguez J. Buján S. Basagaña M. (2017). DNA demethylation of inflammasome-associated genes is enhanced in patients with cryopyrin-associated periodic syndromes. *J. Allergy Clin. Immunol.* 139 202–211.e6 10.1016/j.jaci.2016.05.016 27394913

[B277] Villagra A. Sotomayor E. M. Seto E. (2010). Histone deacetylases and the immunological network: implications in cancer and inflammation. *Oncogene* 29 157–173. 10.1038/onc.2009.334 19855430

[B278] Vladimer G. I. Weng D. Paquette S. W. Vanaja S. K. Rathinam V. A. Aune M. H. (2012). The NLRP12 inflammasome recognizes Yersinia pestis. *Immunity* 37 96–107. 10.1016/j.immuni.2012.07.006 22840842 PMC3753114

[B279] von Moltke J. Trinidad N. J. Moayeri M. Kintzer A. F. Wang S. B. van Rooijen N. (2012). Rapid induction of inflammatory lipid mediators by the inflammasome in vivo. *Nature* 490 107–111. 10.1038/nature11351 22902502 PMC3465483

[B280] Voronov E. Dayan M. Zinger H. Gayvoronsky L. Lin J.-P. Iwakura Y. (2006). IL-1B-deficient mice are resistant to induction of experimental SLE. *Eur. Cytokine Netw.* 17 109–116. 16840029

[B281] Waddington C. H. (1956). *Principles of Embryology.* London: George Allen & Unwin, Ltd.

[B282] Walsh C. P. Chaillet J. R. Bestor T. H. (1998). Transcription of IAP endogenous retroviruses is constrained by cytosine methylation. *Nat. Genet.* 20 116–117. 10.1038/2413 9771701

[B283] Wan L. Yuan X. Liu M. Xue B. (2018). miRNA-223-3p regulates NLRP3 to promote apoptosis and inhibit proliferation of hep3B cells. *Exp. Ther. Med.* 15 2429–2435. 10.3892/etm.2017.5667 29467847 PMC5792760

[B284] Wang F. Zhang X. Yan Y. Zhu X. Yu J. Ding Y. (2017). FBX8 is a metastasis suppressor downstream of miR-223 and targeting mTOR for degradation in colorectal carcinoma. *Cancer Lett.* 388 85–95. 10.1016/j.canlet.2016.11.031 27916606

[B285] Wang G. Gu Y. Xu N. Zhang M. Yang T. (2018). Decreased expression of miR-150, miR146a and miR424 in type 1 diabetic patients: association with ongoing islet autoimmunity. *Biochem. Biophys. Res. Commun.* 498 382–387. 10.1016/j.bbrc.2017.06.196 28733034

[B286] Wang H. Luo Q. Feng X. Zhang R. Li J. Chen F. (2018). NLRP3 promotes tumor growth and metastasis in human oral squamous cell carcinoma. *BMC Cancer* 18:500. 10.1186/s12885-018-4403-9 29716544 PMC5930757

[B287] Wang H. Peng W. Ouyang X. Li W. Dai Y. (2012). Circulating microRNAs as candidate biomarkers in patients with systemic lupus erythematosus. *Transl. Res.* 160 198–206. 10.1016/j.trsl.2012.04.002 22683424

[B288] Wang H. Wang Y. Du Q. Lu P. Fan H. Lu J. (2016). Inflammasome-independent NLRP3 is required for epithelial-mesenchymal transition in colon cancer cells. *Exp. Cell Res.* 342 184–192. 10.1016/j.yexcr.2016.03.009 26968633

[B289] Wang J. Lin D. Peng H. Shao J. Gu J. (2014). Cancer-derived immunoglobulin G promotes LPS-induced proinflammatory cytokine production via binding to TLR4 in cervical cancer cells. *Oncotarget* 5 9727. 25179302 10.18632/oncotarget.2359 PMC4259433

[B290] Wang Y. Han Z. Fan Y. Zhang J. Chen K. Gao L. (2017). MicroRNA-9 Inhibits NLRP3 inflammasome activation in human atherosclerosis inflammation cell models through the JAK1/STAT signaling pathway. *Cell. Physiol. Biochem.* 41 1555–1571. 10.1159/000470822 28334721

[B291] Watson P. R. Gautier A. V. Paulin S. M. Bland A. P. Jones P. W. Wallis T. S. (2000). *Salmonella enterica* serovars Typhimurium and Dublin can lyse macrophages by a mechanism distinct from apoptosis. *Infect. Immun.* 68 3744–3747. 10816540 10.1128/iai.68.6.3744-3747.2000 PMC97671

[B292] Wei L.-J. Li J.-A. Bai D.-M. Song Y. (2018). miR-223-RhoB signaling pathway regulates the proliferation and apoptosis of colon adenocarcinoma. *Chem. Biol. Interact.* 289 9–14. 10.1016/j.cbi.2018.04.016 29660302

[B293] Wei M. Wang L. Wu T. Xi J. Han Y. Yang X. (2016). NLRP3 activation was regulated by DNA methylation modification during *Mycobacterium tuberculosis* Infection. *Biomed Res. Int.* 2016:4323281. 10.1155/2016/4323281 27366746 PMC4913066

[B294] Wei Q. Mu K. Li T. Zhang Y. Yang Z. Jia X. (2014). Deregulation of the NLRP3 inflammasome in hepatic parenchymal cells during liver cancer progression. *Lab. Invest.* 94 52–62. 10.1038/labinvest.2013.126 24166187

[B295] Wen H. Gris D. Lei Y. Jha S. Zhang L. Huang M. T. (2011). Fatty acid-induced NLRP3-ASC inflammasome activation interferes with insulin signaling. *Nat. Immunol.* 12 408–415. 10.1038/ni.2022 21478880 PMC4090391

[B296] Williams K. L. Lich J. D. Duncan J. A. Reed W. Rallabhandi P. Moore C. (2005). The CATERPILLER protein monarch-1 is an antagonist of toll-like receptor-, tumor necrosis factor alpha-, and *Mycobacterium tuberculosis*-induced pro-inflammatory signals. *J. Biol. Chem.* 280 39914–39924. 10.1074/jbc.M502820200 16203735 PMC4422647

[B297] Witola W. H. Mui E. Hargrave A. Liu S. Hypolite M. Montpetit A. (2011). NALP1 influences susceptibility to human congenital toxoplasmosis, proinflammatory cytokine response, and fate of *Toxoplasma gondii*-infected monocytic cells. *Infect. Immun.* 79 756–766. 10.1128/IAI.00898-10 21098108 PMC3028851

[B298] Wongjampa W. Ekalaksananan T. Chopjitt P. Chuerduangphui J. Kleebkaow P. Patarapadungkit N. (2018). Suppression of miR-22, a tumor suppressor in cervical cancer, by human papillomavirus 16 E6 via a p53/miR-22/HDAC6 pathway. *PLoS One* 13:e0206644. 10.1371/journal.pone.0206644 30379969 PMC6209303

[B299] Wu L. Li H. Jia C. Y. Cheng W. Yu M. Peng M. (2012). MicroRNA-223 regulates FOXO1 expression and cell proliferation. *FEBS Lett.* 586 1038–1043. 10.1016/j.febslet.2012.02.050 22569260

[B300] Wu M. F. Chen S. T. Yang A. H. Lin W. W. Lin Y. L. Chen N. J. (2013). CLEC5A is critical for dengue virus-induced inflammasome activation in human macrophages. *Blood* 121 95–106. 10.1182/blood-2012-05-430090 23152543

[B301] Xia S.-S. Zhang G.-J. Liu Z.-L. Tian H.-P. He Y. Meng C.-Y. (2017). MicroRNA-22 suppresses the growth, migration and invasion of colorectal cancer cells through a Sp1 negative feedback loop. *Oncotarget* 8:36266. 10.18632/oncotarget.16742 28422727 PMC5482653

[B302] Xie Q. Wei M. Zhang B. Kang X. Liu D. Zheng W. (2018). MicroRNA33 regulates the NLRP3 inflammasome signaling pathway in macrophages. *Mol. Med. Rep.* 17 3318–3327. 10.3892/mmr.2017.8224 29257274

[B303] Xie Z. Huang G. Wang Z. Luo S. Zheng P. Zhou Z. (2018). Epigenetic regulation of Toll-like receptors and its roles in type 1 diabetes. *J. Mol. Med.* 96 741–751. 10.1007/s00109-018-1660-7 30003291

[B304] Xin M. Qiao Z. Li J. Liu J. Song S. Zhao X. (2016). miR-22 inhibits tumor growth and metastasis by targeting ATP citrate lyase: evidence in osteosarcoma, prostate cancer, cervical cancer and lung cancer. *Oncotarget* 7 44252–44265. 10.18632/oncotarget.10020 27317765 PMC5190093

[B305] Xu H. Yang J. Gao W. Li L. Li P. Zhang L. (2014). Innate immune sensing of bacterial modifications of Rho GTPases by the Pyrin inflammasome. *Nature* 513 237–241. 10.1038/nature13449 24919149

[B306] Yan Y. Lu K. Ye T. Zhang Z. (2019). MicroRNA-223 attenuates LPS-induced inflammation in an acute lung injury model via the NLRP3 inflammasome and TLR4/NF-κB signaling pathway via RHOB. *Int. J. Mol. Med.* 43 1467–1477. 10.3892/ijmm.2019.4075 30747229 PMC6365085

[B307] Yang C. A. Chiang B. L. (2015). Inflammasomes and human autoimmunity: a comprehensive review. *J. Autoimmun.* 61 1–8. 10.1016/j.jaut.2015.05.001 26005048

[B308] Yang J. Zhao Y. Shi J. Shao F. (2013). Human NAIP and mouse NAIP1 recognize bacterial type III secretion needle protein for inflammasome activation. *Proc. Natl. Acad. Sci. U.S.A.* 110 14408–14413. 10.1073/pnas.1306376110 23940371 PMC3761597

[B309] Yang Q. Yu C. Yang Z. Wei Q. Mu K. Zhang Y. (2014). Deregulated NLRP3 and NLRP1 inflammasomes and their correlations with disease activity in systemic lupus erythematosus. *J. Rheumatol.* 41 444–452. 10.3899/jrheum.130310 24334646

[B310] Yao Y. Wang J. B. Xin M. M. Li H. Liu B. Wang L. L. (2016). Balance between inflammatory and regulatory cytokines in systemic lupus erythematosus. *Genet. Mol. Res.* 15:gmr7626. 10.4238/gmr.15027626 27323066

[B311] Zaki M. H. Boyd K. L. Vogel P. Kastan M. B. Lamkanfi M. Kanneganti T. D. (2010). The NLRP3 inflammasome protects against loss of epithelial integrity and mortality during experimental colitis. *Immunity* 32 379–391. 10.1016/j.immuni.2010.03.003 20303296 PMC2982187

[B312] Zaki M. H. Vogel P. Malireddi R. K. Body-Malapel M. Anand P. K. Bertin J. (2011). The NOD-like receptor NLRP12 attenuates colon inflammation and tumorigenesis. *Cancer Cell* 20 649–660. 10.1016/j.ccr.2011.10.022 22094258 PMC3761879

[B313] Zhang G. Xia S. Tian H. Liu Z. Zhou T. (2012). Clinical significance of miR-22 expression in patients with colorectal cancer. *Med. Oncol.* 29 3108–3112. 10.1007/s12032-012-0233-9 22492279

[B314] Zhang H. Tang J. Li C. Kong J. Wang J. Wu Y. (2015a). MiR-22 regulates 5-FU sensitivity by inhibiting autophagy and promoting apoptosis in colorectal cancer cells. *Cancer Lett.* 356 781–790. 10.1016/j.canlet.2014.10.029 25449431

[B315] Zhang H. Wang S. Huang Y. Wang H. Zhao J. Gaskin F. (2015b). Myeloid-derived suppressor cells are proinflammatory and regulate collagen-induced arthritis through manipulating Th17 cell differentiation. *Clin. Immunol.* 157 175–186. 10.1016/j.clim.2015.02.001 25680967 PMC4657752

[B316] Zhao Y. Yang J. Shi J. Gong Y. N. Lu Q. Xu H. (2011). The NLRC4 inflammasome receptors for bacterial flagellin and type III secretion apparatus. *Nature* 477 596–600. 10.1038/nature10510 21918512

[B317] Zhi H. Yuan N. Wu J. P. Lu L. M. Chen X. Y. Wu S. K. (2018). MicroRNA-21 attenuates BDE-209-induced lipid accumulation in THP-1 macrophages by downregulating Toll-like receptor 4 expression. *Food Chem. Toxicol.* 125 71–77. 10.1016/j.fct.2018.12.044 30597220

[B318] Zhong F. L. Robinson K. Teo D. E. T. Tan K. Y. Lim C. Harapas C. R. (2018). Human DPP9 represses NLRP1 inflammasome and protects against autoinflammatory diseases via both peptidase activity and FIIND domain binding. *J. Biol. Chem.* 293 18864–18878. 10.1074/jbc.RA118.004350 30291141 PMC6295727

[B319] Zhou C. C. Yang X. Hua X. Liu J. Fan M. B. Li G. Q. (2016). Hepatic NAD+ deficiency as a therapeutic target for non-alcoholic fatty liver disease in ageing. *Br. J. Pharmacol.* 173 2352–2368. 10.1111/bph.13513 27174364 PMC4945761

[B320] Zhou R. Tardivel A. Thorens B. Choi I. Tschopp J. (2010). Thioredoxin-interacting protein links oxidative stress to inflammasome activation. *Nat. Immunol.* 11 136–140. 10.1038/ni.1831 20023662

[B321] Zhou R. Yazdi A. S. Menu P. Tschopp J. (2011). A role for mitochondria in NLRP3 inflammasome activation. *Nature* 469 221–225. 10.1038/nature09663 21124315

[B322] Zhou Y. Lu M. Du R. H. Qiao C. Jiang C. Y. Zhang K. Z. (2016). MicroRNA-7 targets Nod-like receptor protein 3 inflammasome to modulate neuroinflammation in the pathogenesis of Parkinson’s disease. *Mol. Neurodegener.* 11:28. 10.1186/s13024-016-0094-3 27084336 PMC4833896

[B323] Zhou W. Chen C. Chen Z. Liu L. Jiang J. Wu Z. (2018). NLRP3: a novel mediator in cardiovascular disease. *J. Immunol. Res* 2018:5702103. 10.1155/2018/5702103 29850631 PMC5911339

[B324] Zhu S. Ding S. Wang P. Wei Z. Pan W. Palm N. W. (2017). Nlrp9b inflammasome restricts rotavirus infection in intestinal epithelial cells. *Nature* 546 667–670. 10.1038/nature22967 28636595 PMC5787375

[B325] Zhu S. Pan W. Song X. Liu Y. Shao X. Tang Y. (2012). The microRNA miR-23b suppresses IL-17-associated autoimmune inflammation by targeting TAB2, TAB3 and IKK-alpha. *Nat. Med.* 18 1077–1086. 10.1038/nm.2815 22660635

[B326] Zhu Z. Zhong S. Shen Z. (2011). Targeting the inflammatory pathways to enhance chemotherapy of cancer. *Cancer Biol. Ther.* 12 95–105. 21623164 10.4161/cbt.12.2.15952

[B327] Zychlinsky A. Prevost M. C. Sansonetti P. J. (1992). *Shigella* flexneri induces apoptosis in infected macrophages. *Nature* 358 167–169. 10.1038/358167a0 1614548

